# Intestine-selective farnesoid X receptor inhibition improves obesity-related metabolic dysfunction

**DOI:** 10.1038/ncomms10166

**Published:** 2015-12-15

**Authors:** Changtao Jiang, Cen Xie, Ying Lv, Jing Li, Kristopher W. Krausz, Jingmin Shi, Chad N. Brocker, Dhimant Desai, Shantu G. Amin, William H. Bisson, Yulan Liu, Oksana Gavrilova, Andrew D. Patterson, Frank J. Gonzalez

**Affiliations:** 1 Laboratory of Metabolism, Center for Cancer Research, National Cancer Institute, National Institutes of Health, Bethesda, Maryland 20892, USA; 2 Department of Physiology and Pathophysiology, School of Basic Medical Sciences, Peking University, and the Key Laboratory of Molecular Cardiovascular Science, Ministry of Education, Beijing 100191, China; 3 Department of Gastroenterology, Peking University People's Hospital, Beijing 100044, China; 4 Department of Pharmacology, College of Medicine, The Pennsylvania State University, Hershey, Pennsylvania 17033, USA; 5 Department of Environmental and Molecular Toxicology, Oregon State University, Corvallis, Oregon 97331, USA; 6 Mouse Metabolism Core Laboratory, National Institute of Diabetes and Digestive and Kidney Diseases, National Institutes of Health, Bethesda, Maryland 20892, USA; 7 Department of Veterinary and Biomedical Sciences and the Center for Molecular Toxicology and Carcinogenesis, The Pennsylvania State University, University Park, Pennsylvania 16802, USA

## Abstract

The farnesoid X receptor (FXR) regulates bile acid, lipid and glucose metabolism. Here we show that treatment of mice with glycine-β-muricholic acid (Gly-MCA) inhibits FXR signalling exclusively in intestine, and improves metabolic parameters in mouse models of obesity. Gly-MCA is a selective high-affinity FXR inhibitor that can be administered orally and prevents, or reverses, high-fat diet-induced and genetic obesity, insulin resistance and hepatic steatosis in mice. The high-affinity FXR agonist GW4064 blocks Gly-MCA action in the gut, and intestine-specific *Fxr*-null mice are unresponsive to the beneficial effects of Gly-MCA. Mechanistically, the metabolic improvements with Gly-MCA depend on reduced biosynthesis of intestinal-derived ceramides, which directly compromise beige fat thermogenic function. Consequently, ceramide treatment reverses the action of Gly-MCA in high-fat diet-induced obese mice. We further show that FXR signalling in ileum biopsies of humans positively correlates with body mass index. These data suggest that Gly-MCA may be a candidate for the treatment of metabolic disorders.

## Abstract (web summary)

The nuclear farnesoid X receptor (FXR) is activated by bile acids and influences energy metabolism. Here, the authors report a small molecule inhibitor of FXR, glycine-ß-muricholic acid, which inhibits FXR in the intestine and improves metabolic homeostasis by repressing intestinal ceramide synthesis.

## Graphical abstract

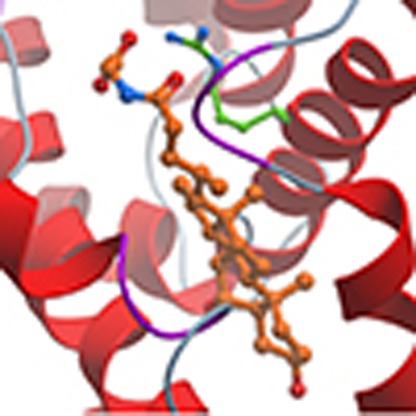

Obesity is associated with chronic diseases such as type 2 diabetes mellitus, cardiovascular diseases, hepatosteatosis and cancer^1^. A chronic imbalance between energy intake and energy expenditure causes obesity for which there is no safe and effective drug therapy^2^. Bariatric surgery, such as Roux-en-Y gastric bypass and vertical sleeve gastrectomy, are among the most effective surgical treatments for obesity^3^,^4^.

Farnesoid X receptor (FXR, NR1H4), a member of the nuclear receptor superfamily of transcription factors^5^, is an important sensor and regulator of bile acid, lipid and glucose metabolism^6^. FXR agonists obeticholic acid and fexaramine were found to improve metabolic profiles in obese mouse models^7^,^8^. Others reported that FXR whole-body knockout mice on a high-fat diet (HFD) displayed metabolic improvement^4^,^9^,^10^. These results suggest that FXR has complex roles in the pathogenesis of metabolic dysfunction, and its activity in liver, intestine, adipose tissue and kidney, could possibly exert different effects on metabolism; thus specific inhibition of FXR needs to be evaluated. Recent studies in which mice on a HFD were treated with the gut microbiota-modifying agent tempol or antibiotics, suggested that inhibition of intestinal FXR signalling could be of benefit in amelioration of obesity, insulin resistance and non-alcoholic fatty liver disease^10^,^11^. This led to the search for a compound that could inhibit FXR signalling specifically in the intestine.

Here a new orally available, small molecule, intestine-specific FXR inhibitor, glycine-β-muricholic acid (Gly-MCA), a bile acid that is not hydrolyzed by gut bacterial bile salt hydrolase (BSH), was developed. Oral administration of Gly-MCA to mice prevents and treats diet-induced and genetic obesity, along with insulin resistance and hepatic steatosis without systemic, hepatic or intestinal toxicities. Specific inhibition of a novel intestinal FXR-ceramide axis produced striking metabolic improvement after Gly-MCA treatment. Mechanistic studies revealed that FXR regulates genes involved in ceramide synthesis, and that ceramides mediate the metabolic effects of Gly-MCA. Specific inhibition of intestinal FXR may be a reasonable therapeutic strategy for the treatment of human metabolic disorders.

## Results

### FXR signalling in obese human intestine

**Figure 1:**
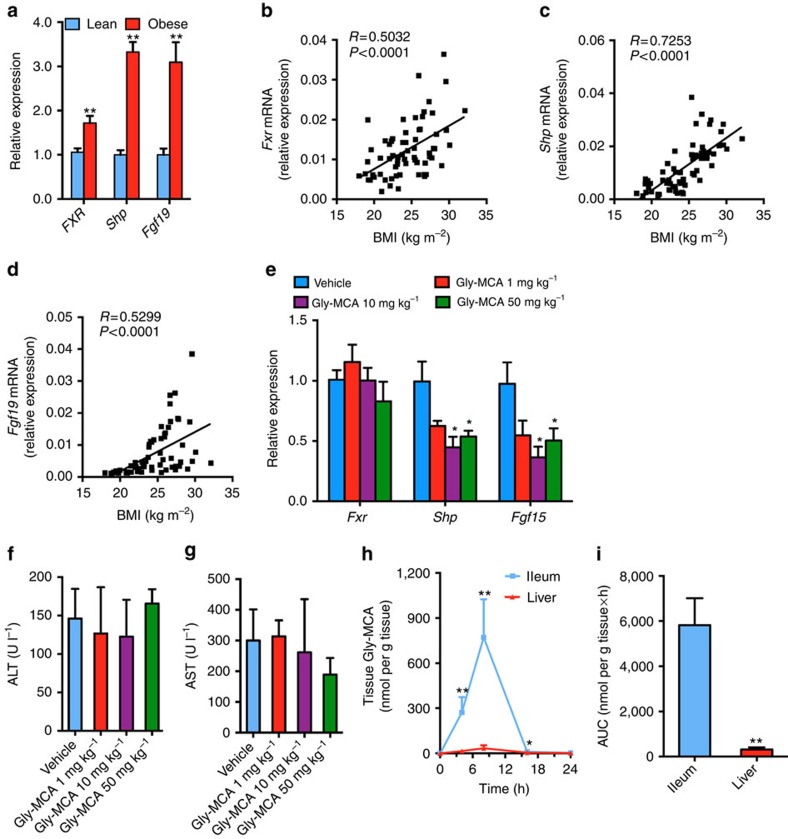
Gly-MCA is an FXR inhibitor. (**a**) mRNA levels of FXR target genes in lean (*n*=36) and obese (*n*=30) human ileum biopsies. Expression was normalized to 18S RNA. Data are presented as mean±s.e.m. Two-tailed Student's *t*-test. **P<0.01 compared with healthy humans (lean). (**b**–**d**) Correlation of *Fxr* (**b**), *Shp* (**c**) and *Fgf19* (**d**) mRNA expressions in human ileum biopsies with body mass index (BMI). *n*=66 total individuals. Expression was normalized to 18S RNA. (**e**) *Fxr*, *Shp* and *Fgf15* mRNA levels in the ileum of mice after 24 h treatment with Gly-MCA. Expression was normalized to 18S RNA. Vehicle group *n*=5, Gly-MCA group *n*=6. Data are presented as mean±s.d. Two-tailed Student's *t*-test. *P<0.05 compared with vehicle. (**f**,**g**) Serum ALT (**f**) and AST (**g**) levels of mice after 24 h treatment with Gly-MCA. Vehicle group *n*=5, Gly-MCA group *n*=6. Data are presented as mean±s.d. Two-tailed Student's *t*-test. (**h**) Ileum and liver Gly-MCA levels. Expression was normalized to 18S RNA. *n*=5 mice per group. Data are presented as mean±s.d. Two-tailed Student's *t*-test. *P<0.05, **P<0.01 compared with liver Gly-MCA levels. (**i**) Tissue Gly-MCA levels as area under the curve (AUC) at the indicated time points 24 h post Gly-MCA (10 mg kg^−1^) treatment. *n*=5 mice per group. Data are presented as mean±s.d. Two-tailed Student's *t*-test. **P<0.01 compared with liver Gly-MCA levels.

FXR signalling was activated in distal ileum biopsies from obese individuals as indicated by an increase in mRNAs encoded by *Fxr* and the FXR target genes small heterodimer partner (*Shp*) and fibroblast growth factor 19 (*Fgf19*; Fig. 1a). Ileal mRNA levels for *Fxr, Shp* and *Fgf19* were also positively correlated with body mass index (BMI; Fig. 1b–d). Thus, suppression of intestinal FXR signalling might be a novel therapeutic target. However, antagonism of FXR as a therapeutic strategy has not been considered due to the potential for cholestasis. Recently, tauro-β-muricholic acid (T-β-MCA) was reported to antagonize FXR signalling in the intestine^10^,^12^ and this inhibition was correlated with improved metabolic function^10^. However, T-β-MCA is rapidly hydrolyzed by gut bacterial BSH, and thus cannot reach concentrations that inhibit gut-specific FXR signalling *in vivo*. This led to the search for small-molecule inhibitors of FXR to treat metabolic dysfunction.

### Identification of an intestine-selective FXR inhibitor

Molecular modelling^13^ was carried out to find derivatives of T-β-MCA that could inhibit FXR leading to the identification of Gly-MCA as inhibitor or possible direct antagonist of FXR (Supplementary Fig. 1a–f and Table 1). To validate inhibition of FXR by Gly-MCA, the HEK293T cell line was transiently co-transfected with a chimeric receptor construct and reporter gene and treated with increasing concentrations of the FXR agonist GW4064 in the presence of vehicle, Gly-MCA, deoxycholic acid (DCA), T-β-MCA, T-α-MCA, β-MCA or α-MCA. Gly-MCA substantially inhibited the FXR transactivation activity induced by GW4064 (Supplementary Fig. 2a). In the human intestinal cell line, Caco-2 cells, 100 μM of the FXR agonist chenodeoxycholic acid (CDCA) caused a fourfold increase in mRNA levels for theFXR target gene *Shp*, while Gly-MCA inhibited the induction of *Shp* mRNA by CDCA (Supplementary Fig. 2b). Similarly, induction of the FXR target genes *Shp* and *Fgf19* by GW4064 were blocked by Gly-MCA (Supplementary Fig. 2c,d). To determine whether Gly-MCA would be stable in the gut, it was subjected to faecal BSH enzyme activity derived from the gut microbiota. Indeed, Gly-MCA was resistant to hydrolysis by BSH, in contrast to T-β-MCA that was rapidly hydrolyzed (Supplementary Fig. 2e). The question arose as to whether Gly-MCA inhibited FXR signalling *in vivo*. To reflect its potential clinical use, bacon-flavoured dough pills were produced for the oral administration of Gly-MCA (dose of 0, 1, 10 and 50 mg kg^−1^). The *Shp* and *Fgf15* (*Fgf19* in human) mRNAs in ileum were decreased to 0.447±0.087 (*P*=0.019) and 0.364±0.089 (*P*=0.015), respectively, at a dose of 10 mg kg^−1^ Gly-MCA for 24 h compared with vehicle (Fig. 1e). Moreover, the higher dose of Gly-MCA (50 mg kg^−1^) did not further inhibit FXR signalling in ileum (Fig. 1e). Gly-MCA did not cause any liver toxicity at each of the doses compared with vehicle, as revealed by serum aminotransferase (ALT) and aspartate aminotransferase (AST) levels (Fig. 1f,g). Pharmacokinetic analysis further showed that Gly-MCA levels in liver, which were almost undetectable, were much lower than in ileum (Fig. 1h,i). These results indicated that Gly-MCA via oral administration, was not significantly absorbed into liver or was rapidly metabolized in the liver. Conversely, Gly-MCA mainly accumulates in ileum where it can selectively inhibit intestinal FXR signalling. These studies identified Gly-MCA as a novel, potent, stable and orally available intestinal FXR inhibitor.

### Gly-MCA reduces obesity and metabolic syndrome in obese mice

To determine whether inhibition of intestinal FXR could be a therapeutic target for HFD-induced obesity, insulin resistance and hepatic steatosis, and to confirm that this transcription factor is a suitable drug target, mice fed a chow diet or a HFD were orally administered with Gly-MCA. Chronic Gly-MCA treatment did not affect the body weights of mice fed a chow diet (Supplementary Fig. 3a). In contrast, Gly-MCA treatment substantially prevented weight gain produced after a HFD (Supplementary Fig. 3b). The absolute fat mass and the fat/lean mass ratio, measured by NMR, were substantially decreased in HFD-fed mice after 9 weeks of treatment with Gly-MCA compared with vehicle-treated mice (Supplementary Fig. 3c). To explore the mechanism of reduced adiposity in Gly-MCA-treated mice, cumulative food intake and energy expenditure using an energy balance technique (TEE_bal_: food energy intake and body composition change) were measured. Food intake was comparable between the two groups (Supplementary Fig. 3d). Gly-MCA treatment substantially increased energy expenditure, which could contribute to the decreased HFD-induced body weight gain compared with vehicle treatment (Supplementary Fig. 3e).

To clarify the role of Gly-MCA in obesity-related glucose homoeostasis, glucose and insulin tolerance tests (GTT and ITT, respectively) were performed. GTT revealed that Gly-MCA-treated mice displayed substantially reduced blood glucose levels after glucose loading compared with vehicle-treated mice (Supplementary Fig. 3f,g). ITT demonstrated that insulin sensitivity was substantially increased after Gly-MCA treatment (Supplementary Fig. 3h). Fasting serum insulin levels of Gly-MCA-treated mice were lower than vehicle-treated mice (Supplementary Fig. 3i). These results indicated that Gly-MCA prevented mice from HFD-induced obesity and insulin resistance. Liver histology indicated a marked reduction in hepatic lipid droplets with no apparent cholestasis, inflammation and necrosis after Gly-MCA treatment of mice fed a HFD for 9 weeks (Supplementary Fig. 4a), which was reflected by lower liver weights (Supplementary Fig. 4b). Hepatic triglyceride contents decreased to ∼51%, although serum triglyceride levels remained unchanged in mice treated with Gly-MCA compared with vehicle (Supplementary Fig. 4c,d). These results indicated that Gly-MCA treatment protected mice from HFD-induced hepatic steatosis.

To exclude the possibility that the effects of Gly-MCA on metabolic disorders were due to non-specific toxicological effects, serum ALT and AST were measured. Gly-MCA treatment dramatically decreased these enzyme levels compared with vehicle-treated mice (Supplementary Fig. 4e,f), thus indicating that Gly-MCA at the dose employed was not hepatotoxic, but actually decreased HFD-induced hepatic toxicity. To assess potential gastrointestinal toxicity, faecal lipocalin 2 (LCN-2), a sensitive and non-invasive biomarker for intestinal toxicity and inflammation, was measured^14^. Gly-MCA did not increase faecal LCN-2 levels (Supplementary Fig. 4g), indicating that Gly-MCA did not induce intestinal inflammation.

**Figure 2:**
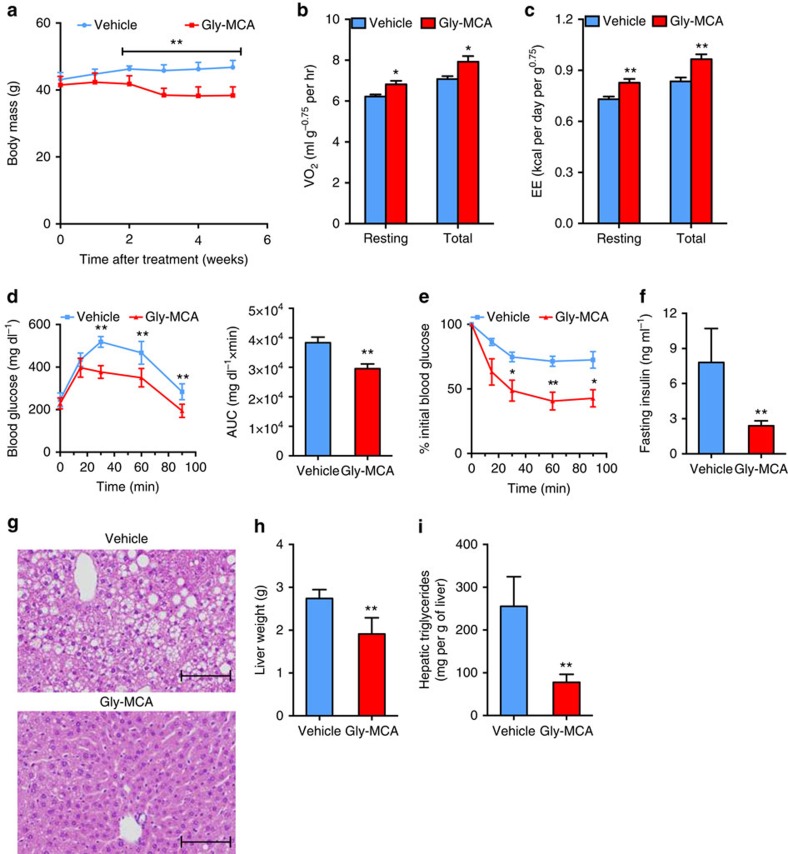
Gly-MCA reverses metabolic dysfunctions in HFD-fed obese mice. (**a**) The growth curves of body weight of established obese mice fed a HFD treated with vehicle and 10 mg kg^−1^ Gly-MCA for 5 weeks. *n*=5 mice per group. Data are presented as mean±s.d. Two-tailed Student's *t*-test. **P<0.01 compared with vehicle treatment. (**b**,**c**) Resting and total O_2_ consumption (**b**), and resting and total energy expenditure (**c**) performed at room temperature in obese mice after 2 weeks of Gly-MCA treatment. *n*=5 mice per group. Data are presented as mean±s.d. Two-tailed Student's *t*-test. *P<0.05, **P<0.01 compared with vehicle treatment. (**d**) Glucose tolerance test (left panel) and the area under the curve (AUC) (right panel) of HFD-induced obese mice treated with vehicle and Gly-MCA for 4 weeks. *n*=5 mice per group. Data are presented as mean±s.d. Two-tailed Student's *t*-test. **P<0.01 compared with vehicle treatment. (**e**) Insulin tolerance test (ITT) of HFD-induced obese mice treated with vehicle and Gly-MCA for 5 weeks. *n*=5 mice per group. Data are presented as mean±s.d. Two-tailed Student's *t*-test. *P<0.05, **P<0.01 compared with vehicle treatment. (**f**) Fasting serum insulin levels. Obese mice fed a HFD were treated with or without Gly-MCA for 5 weeks. *n*=5 mice per group. Data are presented as mean±s.d. Two-tailed Student's *t*-test. **P<0.01 compared with vehicle treatment. (**g**) Representative H&E staining of liver sections. Scale bars, 100 μm. *n*=5 mice per group. (**h**) Liver weights. Obese mice fed a HFD were treated with or without Gly-MCA for 5 weeks. *n*=5 mice per group. Data are presented as mean±s.d. Two-tailed Student's *t*-test. **P<0.01 compared with vehicle treatment. (**i**) Liver triglyceride contents. Obese mice fed a HFD were treated with or without Gly-MCA for 5 weeks. *n*=5 mice per group. Data are presented as mean±s.d. Two-tailed Student's *t*-test. **P<0.01 compared with vehicle treatment.

To evaluate the effects of Gly-MCA on obese mice, C57BL/6N mice were fed a HFD for 12 weeks, and then treated with Gly-MCA. Administration of Gly-MCA daily to obese mice fed a HFD, resulted in reduced body weight as compared with vehicle treatment (Fig. 2a). Fat mass and the fat/lean mass ratio were substantially decreased in Gly-MCA-treated obese mice compared with vehicle-treated mice after 5 weeks of treatment (Supplementary Fig. 5a). Cumulative food intake remained similar between the two groups (Supplementary Fig. 5b). Indirect calorimetry indicated that Gly-MCA treatment significantly increased energy expenditure (Fig. 2b,c). GTT demonstrated that the response to glucose challenge is substantially improved in Gly-MCA-treated obese mice (Fig. 2d). Moreover, ITT revealed that Gly-MCA ameliorated insulin sensitivity (Fig. 2e). Administration of Gly-MCA decreased fasting serum insulin levels of obese mice fed a HFD (Fig. 2f). Liver histology indicated the loss of hepatic lipid droplets with no apparent cholestasis, inflammation and necrosis after Gly-MCA treatment (Fig. 2g). Gly-MCA treatment also reduced liver weights and hepatic triglyceride levels (Fig. 2h,i), and Gly-MCA-treated obese mice displayed reduced serum ALT and AST levels (Supplementary Fig. 5c,d), and faecal LCN-2 levels were similar between Gly-MCA-treated and vehicle-treated mice (Supplementary Fig. 5e). Mice returned to their original weights 4–5 weeks after removal of Gly-MCA (Supplementary Fig. 5f), indicating that Gly-MCA produced reversible weight loss in obese mice. In another model of genetic obesity, leptin receptor-deficient (*db*/*db*) mice, Gly-MCA administration daily for 6 weeks reduced body weight (Supplementary Fig. 6a), fat mass and the fat/lean mass ratio compared with vehicle-treated mice (Supplementary Fig. 6b). Liver histology indicated a significant decrease in hepatic lipid droplets with no observed cholestasis, inflammation and necrosis after Gly-MCA treatment (Supplementary Fig. 6c). Gly-MCA treatment decreased liver weights and hepatic triglyceride contents, whereas serum triglyceride levels were unchanged (Supplementary Fig. 6d–f). Gly-MCA treatment substantially decreased serum ALT and AST levels, while faecal LCN-2 levels were unchanged (Supplementary Fig. 6g–i), thus indicating that Gly-MCA was not toxic to liver and intestine of the *db*/*db* mice, and actually reduced hepatic lipid toxicity in this mouse model.

### Gly-MCA inhibits FXR signalling in ileum, but not liver

**Figure 3:**
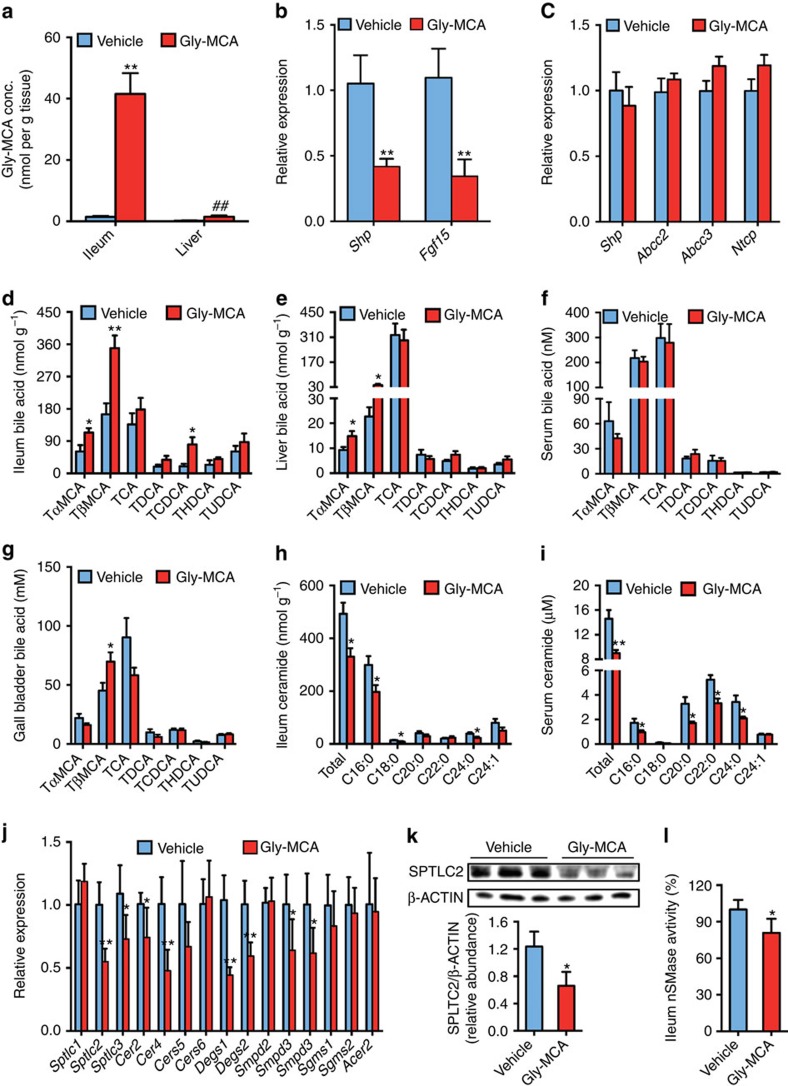
Gly-MCA selectively inhibits intestinal FXR signal and regulates bile acid composition and ceramide metabolism. Mice were treated with or without 10 mg kg^−1^ Gly-MCA once per day. for 9 weeks concurrently on a HFD. (**a**) Gly-MCA levels in ileum and liver. *n*=5 mice per group. Data are presented as mean±s.d. One-way analysis of variance with Tukey's correction. **P<0.01 compared with vehicle-treated mice. ##*P*<0.01 compared with Gly-MCA levels of ileum after Gly-MCA treatment. (**b**,**c**) mRNA levels of FXR target genes in ileum (**b**) and liver (**c**). Expression was normalized to 18S RNA. *n*=5 mice per group. Data are presented as mean±s.d. Two-tailed Student's *t*-test. **P<0.01 compared with vehicle-treated mice. (**d**–**g**) Individual taurine-conjugated bile acids levels in ileum (**d**), liver (**e**), serum (**f**) and gall bladder (**g**). *n*=5 mice per group. Data are presented as mean±s.d. Two-tailed Student's *t*-test. *P<0.05, **P<0.01 compared with vehicle-treated mice. (**h**,**i**) Ileum (**h**) and serum (**i**) ceramides levels. *n*=5 mice per group. Data are presented as mean±s.d. Two-tailed Student's *t*-test. *P<0.05, **P<0.01 compared with vehicle-treated mice. (**j**) mRNA levels of ceramide synthesis- and catabolism-related genes in ileum. Expression was normalized to 18S RNA. *n*=5 mice per group. Data are presented as mean±s.d. Two-tailed Student's *t*-test. *P<0.05, **P<0.01 compared with vehicle-treated mice. (**k**) Western blot analysis of SPTLC2 protein expression in ileum of vehicle- and Gly-MCA-treated mice and quantitation of SPTLC2 expression. SPTLC2 protein levels in ileum of vehicle- and Gly-MCA-treated mice. *n*=3 mice per group. Data are presented as mean±s.d. Two-tailed Student's *t*-test. *P<0.05 compared with vehicle-treated mice. Full western blot image is shown in Supplementary Fig. 17. (**l**) SMPD3 enzyme activities in ileum of vehicle- and Gly-MCA-treated mice. *n*=5 mice per group. Data are presented as mean±s.d. Two-tailed Student's *t*-test. *P<0.05 compared with vehicle-treated mice.

The accumulation of Gly-MCA in the ileum is much greater than in liver in Gly-MCA-treated mice in the different models tested (Fig. 3a, Supplementary Figs 8a and 9a). Gly-MCA treatment inhibited FXR signalling, as revealed by a decrease in *Shp* and *Fgf15* mRNAs in the ileum, but not in the liver of the mice concurrently fed a HFD (Fig. 3b,c). Certain bile acids are known to activate TGR5 and PXR signalling pathways^15^, and therefore the effect of Gly-MCA on TGR5 and PXR signalling pathways was evaluated. Luciferase reporter assays indicated that Gly-MCA failed to activate TGR5 signalling (Supplementary Fig. 7a). Furthermore, the expression of mRNAs encoded by the *Tgr5* and *Glp1* genes in the ileum remained similar after Gly-MCA treatment in the mice fed a HFD (Supplementary Fig. 7b). Gly-MCA treatment did not alter ileum cAMP levels and serum GLP-1 levels of the mice fed a HFD (Supplementary Fig. 7c,d). In addition, PXR mRNAs and PXR target gene mRNAs *Cyp3a11* and *Mdr1* in the ileum were comparable between vehicle- and Gly-MCA-treated mice fed a HFD (Supplementary Fig. 7e). These observations indicated that Gly-MCA had no effects on the *Tgr5*–*Glp1* pathway and PXR signalling, and that Gly-MCA is an intestine-specific FXR inhibitor circumventing the potential adverse outcomes that would result from liver FXR antagonism.

### Gly-MCA reduces intestinal and serum ceramides levels

FXR is the central sensor and regulator of the biosynthesis and enterohepatic recycling of bile acids^16^. Metabolomics analysis revealed that T-α-MCA and T-β-MCA levels of ileum and liver, and T-β-MCA levels of gall bladder were moderately increased, while serum levels of taurine-conjugated bile acids remained unchanged in Gly-MCA-treated mice concurrently fed a HFD compared with vehicle (Fig. 3d–g). Similar results were obtained after Gly-MCA treatment in established obese mice fed a HFD or *db/db* mice (Supplementary Figs 8b–d and 9b–e). Hepatic cholesterol 7α-hydroxylase (CYP7A1) regulates T-α-MCA and T-β-MCA synthesis, and sterol 12α-hydroxylase (CYP8B1) is required for taurocholic acid (TCA) synthesis^17^,^18^. Previous studies demonstrated that the intestinal *Fxr*–*fgf15* signalling pathway regulates *Cyp7a1*, but not *Cyp8b1* (ref. 19). Consistently, *Cyp7a1* mRNA levels were substantially induced after Gly-MCA treatment, but *Cyp8b1* mRNA levels were similar between the two groups, which resulted in the increase of T-α-MCA and T-β-MCA levels (Supplementary Fig. 7f, Fig. 3d). Lipidomics analysis showed that ileum ceramides, especially the most abundant C16:0 ceramide, were substantially reduced, accompanied by lower serum ceramides levels in Gly-MCA-treated mice compared with vehicle-treated mice concurrently fed a HFD (Fig. 3h,i). Similar results were obtained after Gly-MCA treatment in mice made obese with a HFD or *db/db* mice (Supplementary Figs 8e,f and 9f,g). Expression of ceramide synthesis-related gene mRNAs *Sptlc2, Sptlc3*, *Cers2*, *Cers4*, *Degs1*, *Degs2*, *Smpd3* and *Smpd4* were substantially downregulated in ileum of Gly-MCA-treated mice compared with vehicle-treated mice fed a HFD for 9 weeks, but not in liver and white adipose tissue (WAT; Fig. 3j, Supplementary Fig. 7g,h). The mRNAs from ceramide catabolism-related genes, for example, *Sgms1*, *Sgms2*, and *Acer2*, remained similar in ileum, liver and WAT between the two groups (Fig. 3j, Supplementary Fig. 7g,h). *Fgf21* recently emerged as a regulator of energy metabolism and alters ceramides in an adiponectin-dependent manner^20^. However, Gly-MCA did not affect hepatic *Fgf21* mRNA (Supplementary Fig. 7i).

### Intestinal FXR is required for Gly-MCA-decreased ceramides

**Figure 4:**
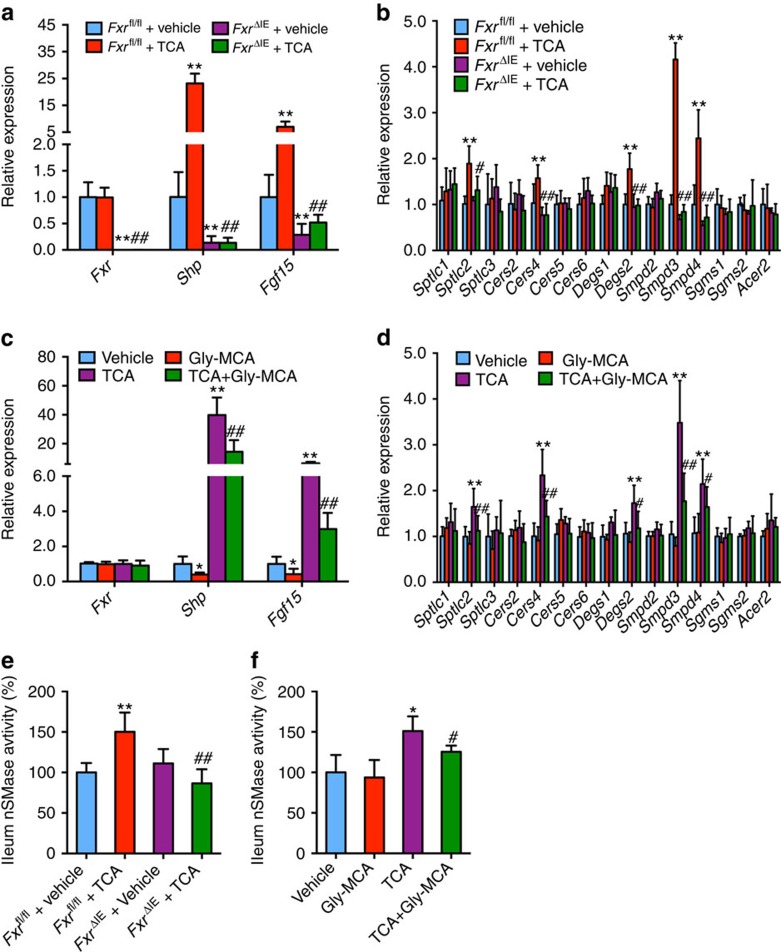
FXR directly regulates ceramide synthesis-related genes. (**a**,**c**) mRNA levels of FXR and FXR target genes in ileum. Expression was normalized to 18S RNA. *n*=5 mice per group. Data are presented as mean±s.d. One-way analysis of variance with Tukey's correction. **P<0.01 compared with vehicle-treated mice. ##*P*<0.01 compared with TCA-treated mice. (**b**,**d**) mRNA levels of ceramide synthesis- and catabolism-related genes in ileum. (**a**,**b**) 6 weeks old *Fxr*^fl/fl^ and *Fxr*^ΔIE^ mice fed a chow diet were treated with or without TCA (400 mg kg^−1^) for 24 h. (**c**,**d**) 6-week-old wild-type mice fed a chow diet were treated with vehicle, Gly-MCA (10 mg kg^−1^), TCA (400 mg kg^−1^), and TCA (400 mg kg^−1^)+Gly-MCA (10 mg kg^−1^) for 24 h. Expression was normalized to 18S RNA. *n*=5 mice per group. Data are presented as mean±s.d. One-way analysis of variance (ANOVA) with Tukey's correction. **P<0.01 compared with vehicle-treated mice. #*P*<0.05, ##*P*<0.01 compared with TCA-treated mice. (**e**) SMPD3 enzyme activities in ileum of *Fxr*^fl/fl^ and *Fxr*^ΔIE^ mice fed a chow diet were treated with or without TCA (400 mg kg^−1^) for 24 h. Expression was normalized to 18S RNA. *n*=5 mice per group. Data are presented as mean±s.d. One-way ANOVA with Tukey's correction. **P<0.01 compared with vehicle-treated mice. ##*P*<0.01 compared with TCA-treated mice. (**f**) SMPD3 enzyme activities in ileum of wild-type mice fed a chow diet were treated with vehicle, Gly-MCA (10 mg kg^−1^), TCA (400 mg kg^−1^), and TCA (400 mg kg^−1^)+Gly-MCA (10 mg kg^−1^) for 24 h. *n*=5 mice per group. Data are presented as mean±s.d. One-way ANOVA with Tukey's correction. *P<0.05 compared with vehicle-treated mice. #*P*<0.05 compared with TCA-treated mice.

To uncover the mechanism by which intestinal FXR regulates ceramide synthesis-related genes, wild-type (WT; *Fxr*^fl/fl^) and intestine-specific *Fxr*-null (*Fxr*^ΔIE^) mice fed a chow diet were treated with the FXR agonist TCA for 24 h. *Fxr* gene expression was almost absent in the ileum of *Fxr*^ΔIE^ mice, and there was no activation of FXR signalling by the natural agonist TCA, as revealed by measurements of the FXR target gene *Shp* and *Fgf15* mRNAs in *Fxr*^ΔIE^ mice in contrast to the robust activation of these genes in *Fxr*^fl/fl^ mice (Fig. 4a). *Sptlc2*, *Cers4*, *Degs2*, *Smpd3* and *Smpd4* mRNAs were also induced in the ileum after TCA treatment in *Fxr*^fl/fl^ mice, but not in *Fxr*^ΔIE^ mice (Fig. 4b). Gly-MCA inhibited TCA-activated FXR signalling in the ileum (Fig. 4c), and further blunted TCA-induced *Sptlc2*, *Cers4*, *Degs2*, *Smpd3* and *Smpd4* mRNAs in the ileum of WT mice (Fig. 4d). Earlier-published intestinal chromatin immunoprecipitation-sequencing data for FXR^21^ revealed FXR binding peaks near the *Sptlc2*, *Degs2* and *Smpd3* genes, and inverted repeat 1 consensus binding motifs for FXR were observed in the FXR-binding peak regions of *Sptlc2* and *Smpd3* genes (http://bit.ly/1W9cvJf). These results suggested that *Sptlc2* and *Smpd3* were potential target genes of FXR in ileum, which was confirmed by promoter–reporter gene studies showing that FXR directly activated the *Sptlc2* and *Smpd3* promoters (C.N.B., unpublished observations). Further, TCA significantly increased the ileal SMPD3 enzyme activities in *Fxr*^fl/fl^ mice, but not in *Fxr*^ΔIE^ mice fed a chow diet (Fig. 4e). The induction of ileal SMPD3 enzyme activities by TCA was significantly inhibited by Gly-MCA treatment in WT mice fed a chow diet (Fig. 4f). Gly-MCA decreased the SPTLC2 protein levels and the SMPD3 enzyme activities in ileum of the mice fed a HFD (Fig. 3k,l). The full western blot for Fig. 3k is found in Supplementary Fig. 17.

**Figure 5:**
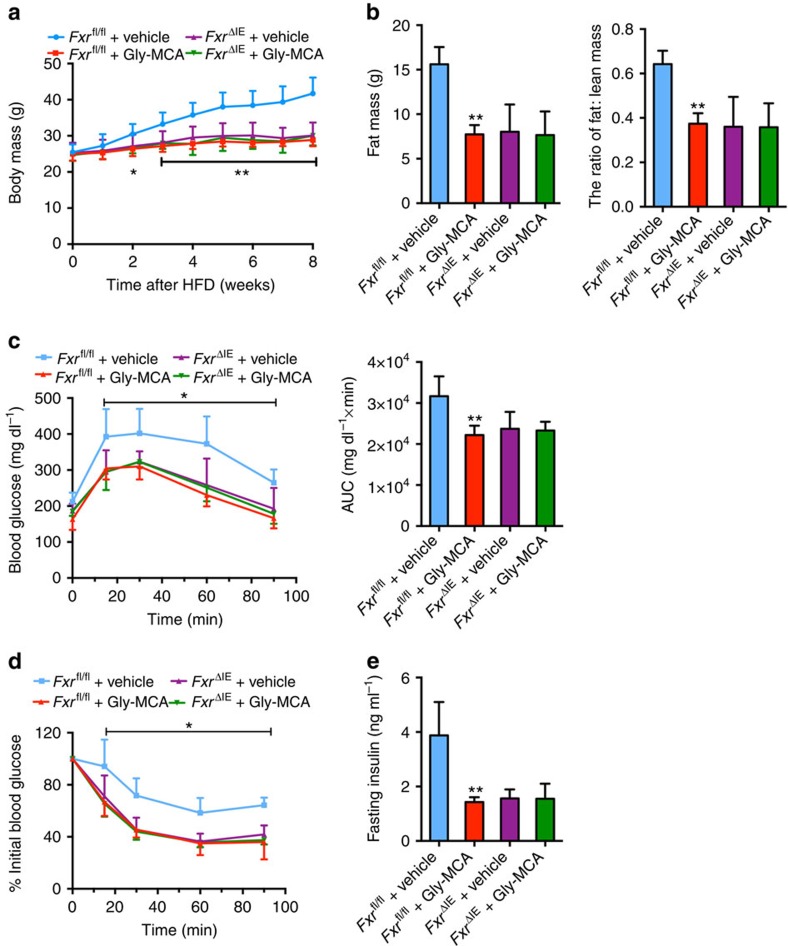
Gly-MCA prevents mice from HFD-induced obesity and insulin resistance via the inhibition of intestinal FXR. (**a**) The growth curves of body weight over the course of 8 weeks, of vehicle- and once per day 10 mg kg^−1^ Gly-MCA-treated *Fxr*^fl/fl^ mice and *Fxr*^ΔIE^ mice, respectively, fed a HFD. *n*=5 mice per group. Data are presented as mean±s.d. One-way analysis of variance (ANOVA) with Tukey's correction. *P<0.05, **P<0.01 compared with vehicle-treated *Fxr*^fl/fl^ mice. (**b**) Fat mass (left panel) and fat mass to lean mass ratio (right panel) of vehicle- and Gly-MCA-treated *Fxr*^fl/fl^ mice and *Fxr*^ΔIE^ mice fed a HFD for 8 weeks. *n*=5 mice per group. Data are presented as mean±s.d. One-way ANOVA with Tukey's correction. **P<0.01 compared with vehicle-treated *Fxr*^fl/fl^ mice. (**c**) Glucose tolerance test (left panel) and the area under the curve (AUC) (right panel) in vehicle- and Gly-MCA-treated *Fxr*^fl/fl^ mice and *Fxr*^ΔIE^ mice after 4 weeks on a HFD. *n*=5 mice per group. Data are presented as mean±s.d. One-way ANOVA with Tukey's correction. *P<0.05 compared with vehicle-treated *Fxr*^fl/fl^ mice. (**d**) Insulin tolerance test (ITT) in vehicle- and Gly-MCA-treated *Fxr*^fl/fl^ mice and *Fxr*^ΔIE^ mice fed a HFD for 5 weeks. *n*=5 mice per group. Data are presented as mean±s.d. One-way ANOVA with Tukey's correction. *P<0.05 compared with vehicle-treated *Fxr*^fl/fl^ mice. (**e**) Fasted serum insulin levels of vehicle- and Gly-MCA-treated *Fxr*^fl/fl^ mice and *Fxr*^ΔIE^ mice fed a HFD for 8 weeks. *n*=5 mice per group. Data are presented as mean±s.d. One-way ANOVA with Tukey's correction. **P<0.01 compared with vehicle-treated *Fxr*^fl/fl^ mice.

To further clarify the role of intestinal FXR in the Gly-MCA-improved metabolic dysfunctions, *Fxr*^fl/fl^ mice and *Fxr*^ΔIE^ mice fed a HFD were treated with vehicle and Gly-MCA. Gly-MCA prevented HFD-induced body weight gain in *Fxr*^fl/f^ mice, but not in *Fxr*^ΔIE^ mice (Fig. 5a). The fat mass and fat/lean mass ratios of Gly-MCA-treated *Fxr*^fl/fl^ mice were substantially decreased to levels similar to those in *Fxr*^ΔIE^ mice (Fig. 5b). *Fxr*^ΔIE^ mice displayed ameliorated glucose intolerance and insulin resistance, and were unresponsive to the metabolic benefits of Gly-MCA treatment (Fig. 5c–e). Liver histology showed that Gly-MCA substantially decreased hepatic lipid droplets in *Fxr*^fl/fl^ mice, but had no effects on *Fxr*^ΔIE^ mice (Supplementary Fig. 10a). The liver weights and hepatic triglyceride contents in Gly-MCA-treated *Fxr*^fl/fl^ mice, and vehicle-treated *Fxr*^ΔIE^ mice, were substantially lower compared with vehicle-treated *Fxr*^fl/fl^ mice (Supplementary Fig. 10b,c). These results indicated that Gly-MCA did not further improve obesity, insulin resistance and hepatic steatosis beyond that was achieved in *Fxr*^ΔIE^ mice.

### Ceramide reverses the beneficial metabolic effects of Gly-MCA

**Figure 6:**
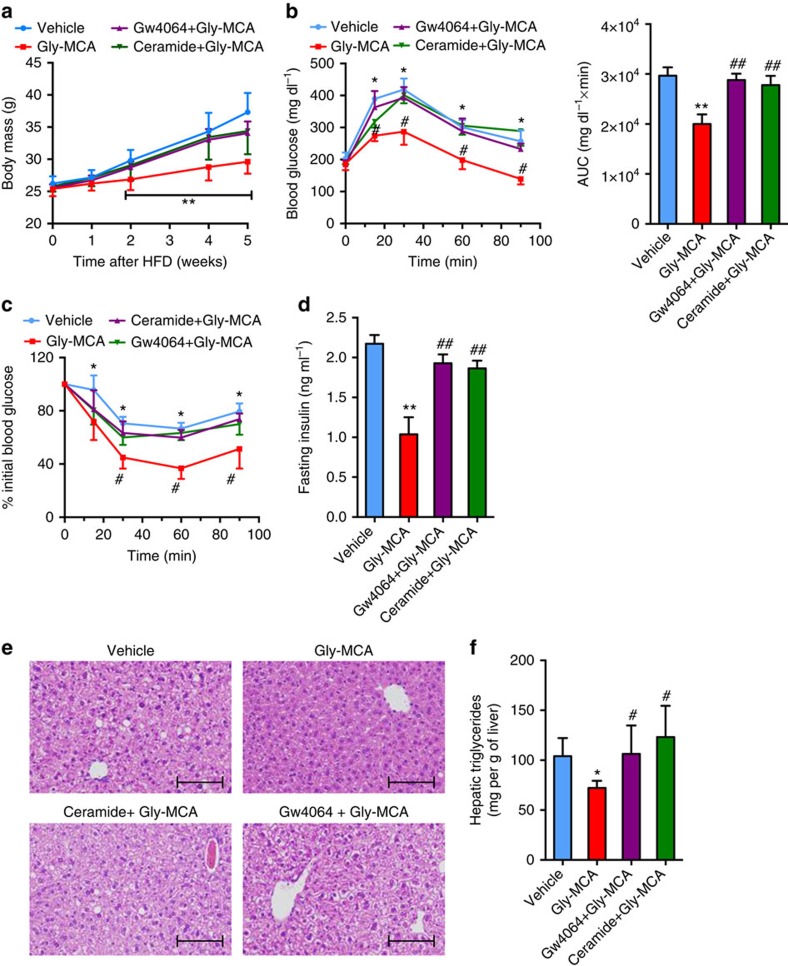
Gly-MCA protects mice from HFD-induced metabolic dysfunctions through inhibition of the intestinal FXR-ceramide axis. (**a**) The growth curves of body weight over the course of 5 weeks, of vehicle-, 10 mg kg^−1^ Gly-MCA-, GW4064+Gly-MCA-, ceramide+Gly-MCA-treated mice, respectively, fed a HFD. *n*=5 mice per group. Data are presented as mean±s.d. One-way analysis of variance (ANOVA) with Tukey's correction. **P<0.01 compared with vehicle-treated mice. (**b**) Glucose tolerance test (left panel) and the area under the curve (AUC) (right panel) in vehicle-, Gly-MCA-, GW4064+Gly-MCA− and ceramide+Gly-MCA-treated mice concurrently fed a HFD for 4 weeks. *n*=5 mice per group. Data are presented as mean±s.d. One-way ANOVA with Tukey's correction. *P<0.05, **P<0.01 compared with vehicle-treated mice. #*P*<0.05, ##*P*<0.01 compared with Gly-MCA-treated mice. (**c**) Insulin tolerance test (ITT) in vehicle-, Gly-MCA-, GW4064+Gly-MCA- and ceramide+Gly-MCA-treated mice concurrently fed a HFD for 5 weeks. *n*=5 mice per group. Data are presented as mean±s.d. One-way ANOVA with Tukey's correction. *P<0.05 compared with vehicle-treated mice. #*P*<0.05 compared with Gly-MCA-treated mice. (**d**) Fasting serum insulin levels. The mice were treated with vehicle, Gly-MCA, GW4064+Gly-MCA and ceramide+Gly-MCA for 5 weeks concurrently on a HFD. *n*=5 mice per group. Data are presented as mean±s.d. One-way ANOVA with Tukey's correction. **P<0.01 compared with vehicle-treated mice. ##*P*<0.01 compared with Gly-MCA-treated mice. (**e**) Representative H&E staining of liver sections. Scale bars: 100 μm. *n*=5 mice per group. (**f**) Liver triglyceride contents. The mice were treated with vehicle, Gly-MCA−, GW4064+Gly-MCA and ceramide+Gly-MCA for 5 weeks concurrently on a HFD. *n*=5 mice per group. Data are presented as mean±s.d. One-way ANOVA with Tukey's correction. *P<0.05 compared with vehicle-treated mice. #*P*<0.05 compared with Gly-MCA-treated mice.

To further evaluate the role of the intestinal FXR–ceramide axis in the Gly-MCA-improved metabolic disorders, the FXR agonist GW4064 (10 mg kg^−1^) or C16:0 ceramide were administered to Gly-MCA-treated mice fed a HFD. Ceramide treatment was found not to affect the pharmacokinetic profile of Gly-MCA (C.X., unpublished observations). GW4064 or ceramide treatment reversed the reduction in body weight gain in Gly-MCA-treated mice (Fig. 6a). The response to glucose and insulin challenge in Gly-MCA-treated mice was blunted after GW4064 or ceramide treatment (Fig. 6b–d). Liver histology indicated that GW4064 or ceramide treatment increased hepatic lipid droplets in Gly-MCA-treated mice (Fig. 6e). Hepatic triglyceride levels were also increased to similar levels in the vehicle group after GW4064 or ceramide treatment of the Gly-MCA-treated mice (Fig. 6f). GW4064 or ceramide treatment reversed ileum and serum ceramides levels, which were decreased by Gly-MCA administration (Supplementary Fig. 11a,b).

### Gly-MCA induces the ‘browning' function of beige fat in scWAT via inhibition of the intestinal FXR-ceramide axis

**Figure 7:**
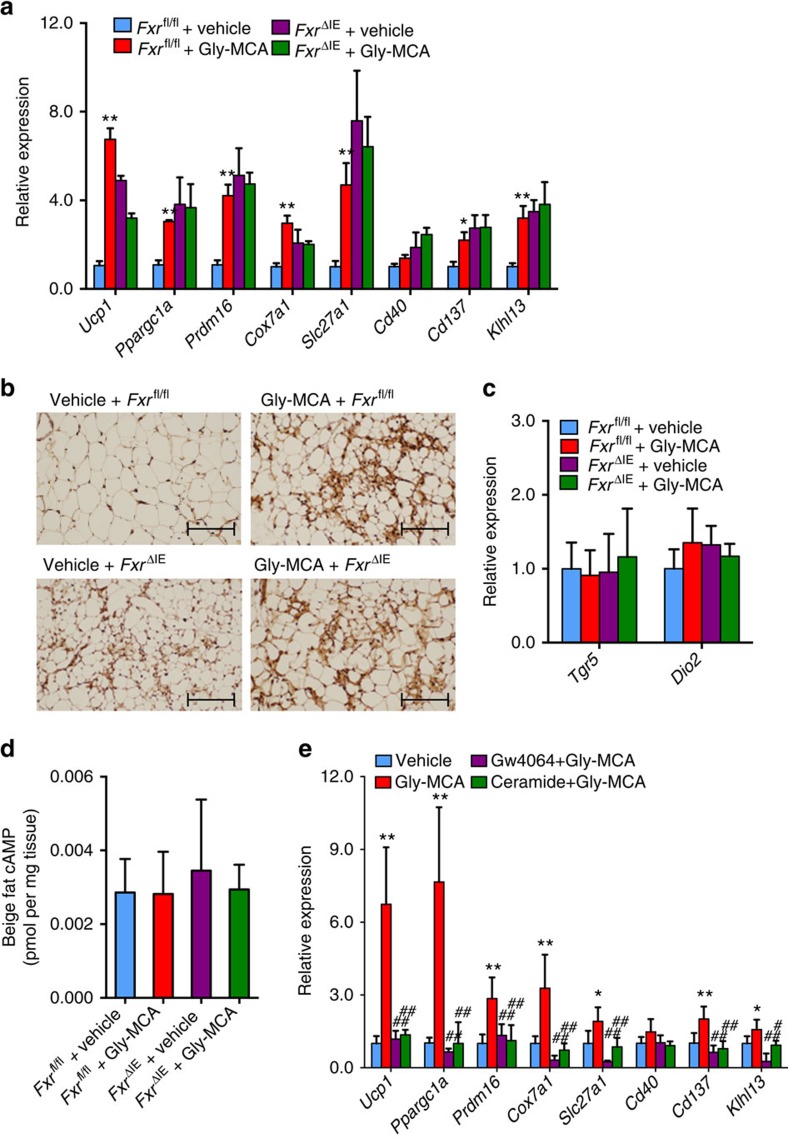
Gly-MCA improves beige fat biogenesis via the inhibition of intestinal FXR-ceramide axis. (**a**) Beige fat thermogenic gene expression in subcutaneous adipose tissue of vehicle- and Gly-MCA-treated *Fxr*^fl/fl^ and *Fxr*^ΔIE^ mice concurrently fed a HFD for 8 weeks. *n*=5 mice per group. Data are presented as mean±s.d. One-way analysis of variance (ANOVA) with Tukey's correction. *P<0.05, **P<0.01 compared with vehicle-treated mice. (**b**) Representative UCP1 immunohistochemistry staining of subcutaneous adipose tissue sections from vehicle- and Gly-MCA-treated *Fxr*^fl/fl^ and *Fxr*^ΔIE^ mice concurrently fed a HFD for 8 weeks. *n*=5 mice per group. (**c**) *Tgr5* and *Dio2* mRNA expression in subcutaneous adipose tissue of vehicle- and Gly-MCA-treated *Fxr*^fl/fl^ and *Fxr*^ΔIE^ mice concurrently fed a HFD for 8 weeks. *n*=5 mice per group. Data are presented as mean±s.d. One-way ANOVA with Tukey's correction. (**d**) The cAMP levels of beige fat in vehicle- and Gly-MCA-treated *Fxr*^fl/fl^ and *Fxr*^ΔIE^ mice concurrently fed a HFD for 8 weeks. *n*=5 mice per group. Data are presented as mean±s.d. One-way ANOVA with Tukey's correction. (**e**) mRNA levels of beige fat thermogenic genes in subcutaneous adipose tissue from vehicle-, Gly-MCA−, GW4064+Gly-MCA− and ceramide+Gly-MCA-treated mice concurrently fed a HFD for 5 weeks. *n*=5 mice per group. Data are presented as mean±s.d. One-way ANOVA with Tukey's correction. *P<0.05, **P<0.01 compared with vehicle-treated mice. #*P*<0.05, ##*P*<0.01 compared with Gly-MCA-treated mice.

The beige fat in subcutaneous WAT (scWAT) and brown fat were found to make an important contribution to the maintenance of energy balance and thermogenesis^22^,^23^,^24^. To explore the mechanism that Gly-MCA increased energy expenditure, mRNA levels for beige fat thermogenic-associated genes were measured. Gly-MCA markedly induced expression of mRNAs encoding *Ucp1*, *Ppargc1a*, *Prdm16*, *Cox7a*, *Slc27a1*, *Cd137*, and *Klhl13* in scWAT of *Fxr*^fl/fl^ mice, but not in *Fxr*^ΔIE^ mice concurrently fed a HFD (Fig. 7a). Immunohistochemistry further confirmed that UCP1^+^ beige adipocytes in scWAT of *Fxr*^fl/fl^ mice were increased after Gly-MCA treatment, whereas no further increase was noted in *Fxr*^ΔIE^ mice treated with Gly-MCA (Fig. 7b). In contrast, Gly-MCA treatment failed to activate brown fat biogenesis related genes in interscapular brown fat of *Fxr*^fl/fl^ mice or *Fxr*^ΔIE^ mice (Supplementary Fig. 12a). Gly-MCA had no effects on the TGR5-DIO2 signalling pathway as revealed by the similar *Tgr5* and *Dio2* mRNA expression levels in beige fat, and brown fat of *Fxr*^fl/fl^ or *Fxr*^ΔIE^ mice between vehicle and Gly-MCA treatment (Fig. 7c, Supplementary Fig. 12b). The β-sympathetic nerve-activated β-adrenergic receptors-cAMP signals exert an important role in the thermogenesis of brown fat and beige fat. Gly-MCA treatment did not alter the cAMP levels in beige fat and brown fat (Fig. 7d, Supplementary Fig. 12c). Moreover, beige fat thermogenic-associated mRNA expression was markedly induced after Gly-MCA treatment at both 29.5 and 5 °C (Supplementary Fig. 13a,b), suggesting that Gly-MCA increased beige fat thermogenesis independent of the activation of β-sympathetic nervous system. Instead, the increase of beige fat thermogenic related mRNAs in Gly-MCA-treated mice concurrently fed a HFD was markedly blunted after GW4064 or ceramide treatment (Fig. 7e). These results implied that inhibition of intestinal FXR-ceramides axis result in improvement of beige fat thermogenesis after Gly-MCA administration.

**Figure 8:**
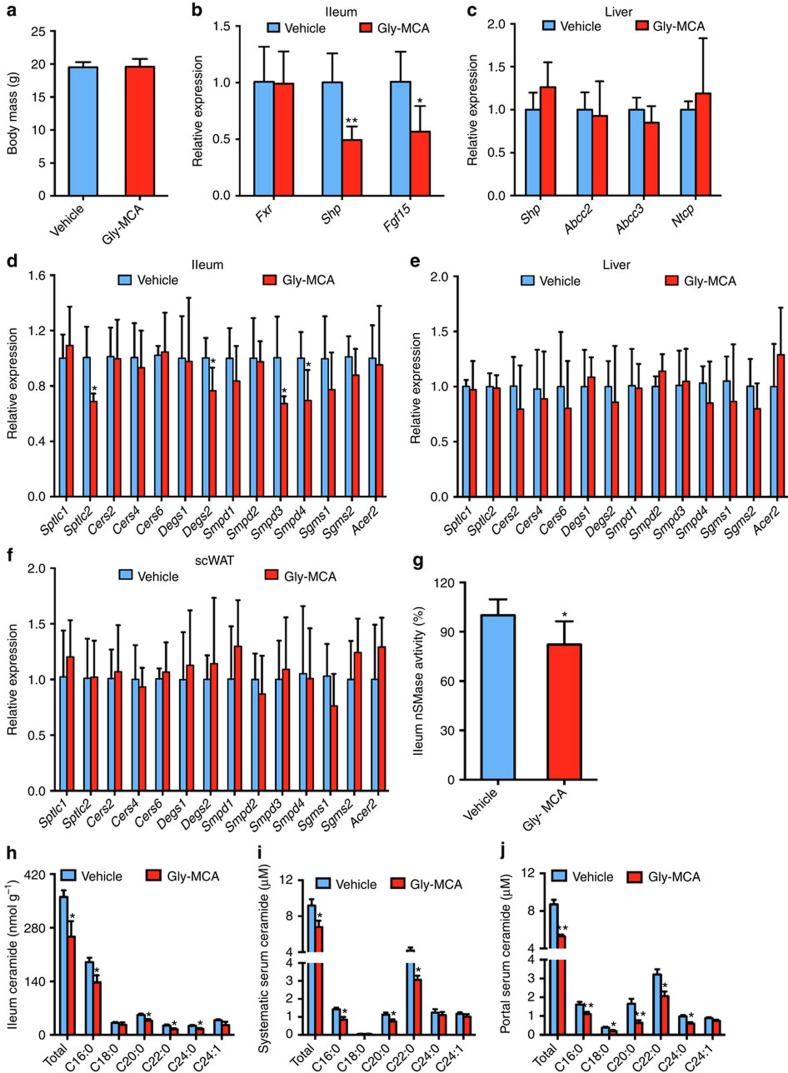
Treatment with Gly-MCA 5 days specifically inhibits intestinal FXR signalling and lowers ceramide metabolism independent of body weight change. Mice on a HFD were treated with once per day 10 mg kg^−1^ Gly-MCA for 5 days. (**a**) Body weights. *n*=5 mice per group. Data are presented as mean±s.d. Two-tailed Student's *t*-test. (**b**,**c**) Ileum and (**b**) liver (**c**) mRNA levels of FXR target genes in ileum. Expression was normalized to 18S RNA. *n*=5 mice per group. Data are presented as mean±s.d. Two-tailed Student's *t*-test. *P<0.05, **P<0.01 compared with vehicle-treated mice. (**d**–**f**) Ceramide synthesis- and catabolism-related gene mRNA levels in ileum (**d**), liver (**e**) and scWAT (**f**). Expression was normalized to 18S RNA. *n*=5 mice per group. Data are presented as mean±s.d. Two-tailed Student's *t*-test. *P<0.05 compared with vehicle-treated mice. (**g**) SMPD3 enzyme activities in ileum. *n*=5 mice per group. Data are presented as mean±s.d. Two-tailed Student's *t*-test. *P<0.05 compared with vehicle-treated mice. (**h**–**j**) Ileum (**h**), portal vein (**i**) and systematic vein (**j**) ceramides levels. *n*=5 mice per group. Data are presented as mean±s.d. Two-tailed Student's *t*-test. *P<0.05, **P<0.01 compared with vehicle-treated mice.

**Figure 9:**
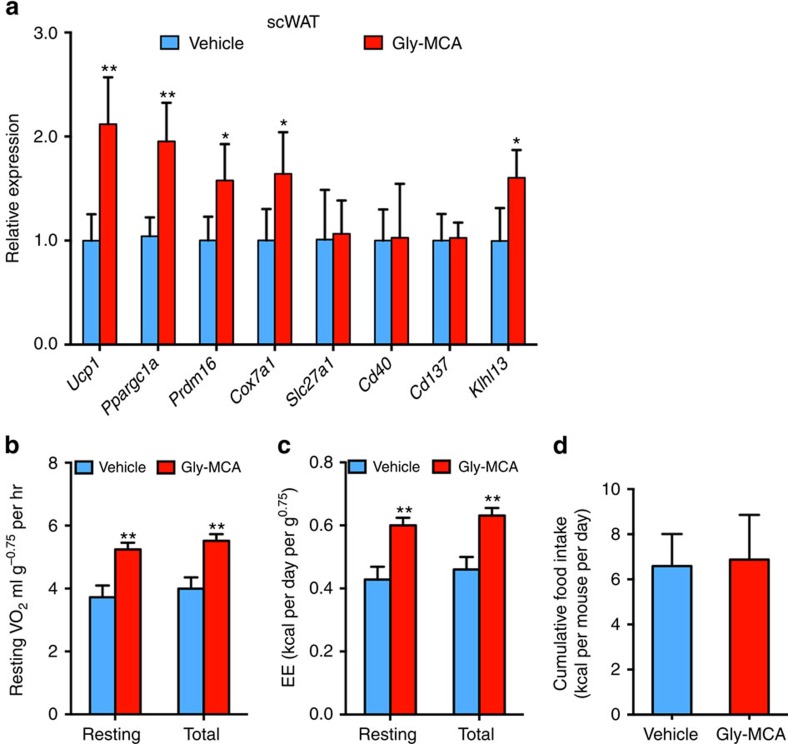
A short-duration 5-day treatment with Gly-MCA specifically increases beige fat thermogenesis and energy expenditure before body weight change. Mice on a HFD were treated with once per day 10 mg kg^−1^ Gly-MCA for 5 days. (**a**) Beige fat thermogenic genes expression of scWAT. Expression was normalized to 18S RNA. *n*=5 mice per group. Data are presented as mean±s.d. Two-tailed Student's *t*-test. *P<0.05, **P<0.01 compared with vehicle-treated mice. (**b**,**c**) Resting and total O_2_ consumption (**b**), and resting and total energy expenditure (**c**) performed at room temperature. *n*=5 mice per group. Data are presented as mean±s.d. Two-tailed Student's *t*-test. **P<0.01 compared with vehicle-treated mice. (**d**) Cumulative food intake per day averaged over 5 days in vehicle- and Gly-MCA-treated obese mice fed a HFD. *n*=5 mice per group. Data are presented as mean±s.d. Two-tailed Student's *t*-test.

To further rule out the possibility that reduced body weight contributed to the observed beneficial metabolic effects of Gly-MCA and to establish a causal relationship between beige fat thermogenesis improvement and body weight loss, mice on a HFD were treated with Gly-MCA for 5 days when no alteration of body weight was noted (Fig. 8a). Gly-MCA substantially inhibited FXR signalling in ileum, but not in liver (Fig. 8b,c). The *Sptlc2*, *Degs2*, *Smpd3* and *Smpd4* mRNAs were decreased in ileum, but not in liver and scWAT after 5 days of Gly-MCA treatment of mice on a HFD (Fig. 8d–f). Gly-MCA significantly lowered SMPD3 enzyme activity in ileum (Fig. 8g). Lipidomics analysis revealed that ileum ceramides were decreased, accompanied by lower portal vein and systematic vein ceramides in Gly-MCA-treated mice compared with vehicle-treated mice (Fig. 8h–j). Gly-MCA-induced expression of *Ucp1*, *Ppargc1a*, *Prdm16*, *Cox7a* and *Klhl13* mRNAs in beige fat, in parallel with an increase of metabolic rate and similar food intake (Fig. 9a–d). These results demonstrated that the improvement of beige fat thermogenesis via inhibition of the intestinal FXR–ceramide axis might be causal rather than due simply to weight loss.

### Ceramides impaired beige fat thermogenesis *in vitro*

**Figure 10:**
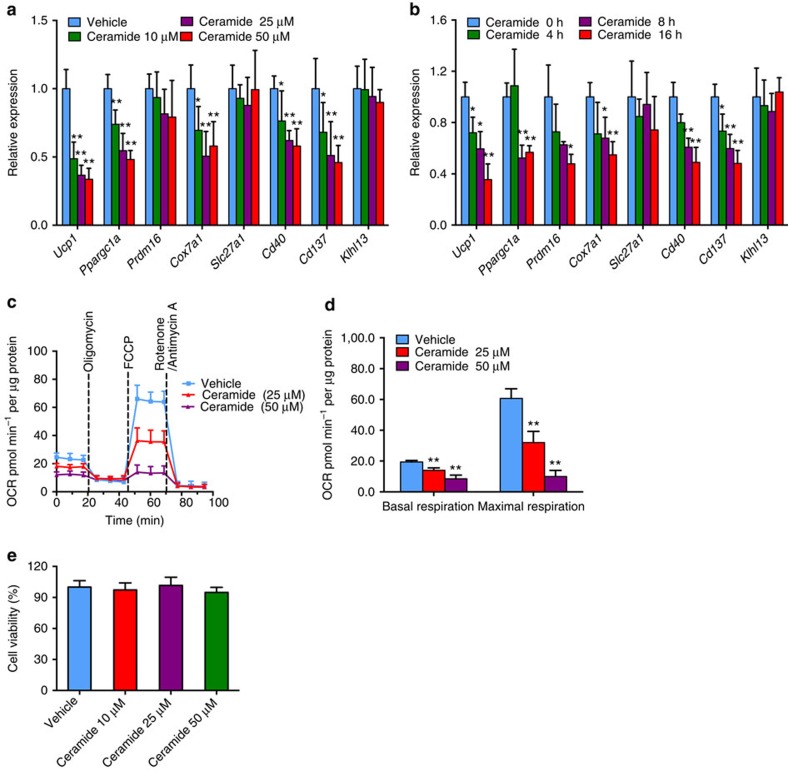
Ceramide compromises ‘browning' function of beige adipocyte. (**a**,**b**) mRNA levels of thermogenic related genes in beige adipocytes after 8 h of indicated doses of ceramide treatment (**a**) or after the indicated time point of 25 μM ceramide treatment (**b**). Six to seven replicates of three separate experiments. Data are presented as mean±s.d. Two-tailed Student's *t*-test. *P<0.05, **P<0.01 compared with control. (**c**) Oxygen consumption rate (OCR) of beige adipocytes after 8 h of indicated concentration ceramide treatment in the presence of 1 μM oligomycin (ATP synthase inhibitor), 1 μM FCCP (uncoupling agent) and 1 μM rotenone (complex I inhibitor) and 1 μM antimycin A (complex III inhibitor). Six replicates of three separate experiments. (**d**) Basal and maximal respiration of beige adipocytes after 8 h of indicated concentration ceramide treatment. Six replicates of three separate experiments. Data are presented as mean±s.d. Two-tailed Student's *t*-test. **P<0.01 compared with control. (**e**) Cell viability after 24 h of the indicated doses of ceramide treatment. (**a**–**e**) Six replicates of three separate experiments. Data are presented as mean±s.d. Two-tailed Student's *t*-test.

To evaluate direct effects of ceramide on beige fat function, induced beige adipocytes^25^ were stimulated by ceramides *in vitro*. Ceramide treatment substantially suppressed expression of *Ucp1*, *Ppargc1a*, *Cox7a*, *Cd40* and *Cd137* mRNAs in beige adipocytes in a dose- and time-dependent manner (Fig. 10a,b). Since mitochondrial respiration rates are used to estimate the ‘browning' function of beige fat^26^, oxygen consumption rate (OCR) analysis was determined. Ceramide treatment markedly decreased basal and maximal respiration of beige adipocytes in a dose-dependent manner (Fig. 10c,d). No cell toxicity was noted at any indicated doses of the ceramides after 24 h treatment (Fig. 10e).

### Gly-MCA reduces serum inflammatory cytokine levels

Gly-MCA was also observed to decrease the levels of mRNAs encoding inflammatory cytokines such as IL-1β, MCP-1, MIP-1α and IL-17, and the adipokine leptin in scWAT of *Fxr*^fl/fl^ mice, but not in *Fxr*^ΔIE^ mice fed a HFD (Supplementary Fig. 14a,b). Consistent with this result, IL-1β, MCP-1, MIP-1α, IL-17 and leptin were lower after Gly-MCA treatment in *Fxr*^fl/fl^ mice, and no further decrease was noted in *Fxr*^ΔIE^ mice treated with Gly-MCA on a HFD (Supplementary Fig. 14c,d). Five days of Gly-MCA treatment decreased *Mcp-1* mRNA levels but not *Il-1β*, and *Mip-1α* mRNA levels that were reduced on Gly-MCA treatment (Supplementary Fig. 14e). Thus, decreased *Mcp-1* expression might contribute to the improvement of insulin sensitivity after Gly-MCA.

### Ceramides levels modulate Gly-MCA improvement of hepatic steatosis

Gly-MCA substantially decreased the expression of mRNAs encoded by fatty acid synthesis-related genes such as *Srebp1c*, *Cidea*, *Fasn* and *Elovl6* in the liver of *Fxr*^fl/fl^ mice, whereas no further decrease was noted in *Fxr*^ΔIE^ mice treated with Gly-MCA (Supplementary Fig. 15a). GW4064 or ceramide treatment reversed the *Srebp1c*, *Cidea*, *Fasn* and *Elovl6* mRNA levels in Gly-MCA-treated mice (Supplementary Fig. 15b). Expression of hepatic mRNAs encoded by triglyceride formation related genes such as *Dgat1* and *Dgat2* remained unchanged after Gly-MCA treatment (Supplementary Fig. 15a,b). These results indicated that Gly-MCA treatment downregulated the *Srebp1c–cidea* pathway through inhibition of the intestinal FXR–ceramide axis.

## Discussion

Increasing evidence points to intestinal FXR as a major regulator of metabolic disorders^4^,^9^,^27^. Notably, constitutive signalling of intestinal FXR as a result of bile acid agonists produced in liver appears to aggravate obesity, insulin resistance and fatty liver, and thus antagonism of intestinal FXR may be a reasonable therapeutic strategy. Gly-MCA was identified as a new orally available, intestinal-selective FXR inhibitor that circumvents the side effects of hepatic FXR inhibition that would result in increased cholestasis as found in *Fxr*-null mice^16^. Gly-MCA prevented and treated metabolic dysfunction resulting from HFD treatment and genetic obesity via the inhibition of intestinal FXR signalling. No inhibition of hepatic FXR signalling was noted and no cholestasis was observed in Gly-MCA-treated mice. Mechanistically, a decrease in intestinal-derived ceramides, as a result of lower ceramide synthesis-related genes by inhibition of FXR, likely mediates the resolution of the HFD-induced obesity and hepatic steatosis by Gly-MCA, although at this time it is uncertain whether ceramides affect insulin resistance. Ceramide injection reversed the effects of Gly-MCA on HFD-fed WT mice, and the associated metabolically favourable phenotype of HFD-fed *Fxr*^ΔIE^ mice, although it should be noted that they have no metabolic abnormalities found in HFD-fed WT mice. FXR was found to directly regulate ceramide metabolism in intestine as revealed by the increase of *Sptlc2* mRNA and the corresponding protein, and the upregulation of *Smpd3* mRNA and enzyme activity in the intestine, and FXR activation of the *Sptlc2* and *Smpd3* promoters. More importantly, the reduced degree of portal vein ceramides (from intestine) was greater than that from the systematic vein ceramides (38% reduced versus 25% reduced for portal vein ceramides levels and systematic vein ceramides levels, respectively). In addition, no alterations were observed for ceramide catabolism-related genes in ileum, liver and WAT. Thus, the serum ceramide changes after Gly-MCA treatment mainly come from intestine metabolism. The site of reduced ceramide synthesis, the intestine and the partial reduction of ceramides through suppression of intestinal FXR signalling, is rather fortuitous, since major reductions in ceramides might cause central nervous system toxicity and skin toxicity since spingolipids are important constituents of these organs^28^,^29^.

Obese humans were found to have increased FXR expression and FXR signalling in the intestine as compared with lean controls. While a cause–effect relationship was not established, these data suggest that since FXR is activated in human obesity, and that gut-specific inhibition may be a promising human therapy. Supporting this view, the bile acid sequestrant colesevelam, a clinically-used anti-diabetes drug, was found to improve glucose homoeostasis partially through inhibition of FXR signalling^30^,^31^. Colesevelam also improved insulin resistance via altering TGR5–GLP-1 signalling in DIO-mice and rats^32^,^33^.

HFD-fed mice given Gly-MCA had improved insulin sensitivity and resolution of fatty liver, and thus the question arises whether these improved metabolic endpoints are the result of lower obesity. However, mice on a HFD for 5 days without any significant weight change had improved insulin resistance. There might be two potential mechanisms to explain this result. First, the increased beige fat function via inhibition of intestinal-derived ceramides might lead to improved insulin resistance in Gly-MCA-treated mice. Supporting this view, beige fat is not only a fat-burning organ but also impacts glucose homoeostasis^24^,^34^. Second, 5 days of Gly-MCA treatment significantly decrease *Mcp-1* mRNA expression in scWAT. Others found that MCP-1 promotes adipose tissue inflammation and insulin resistance^35^,^36^. However, the precise mechanism by which Gly-MCA improved insulin sensitivity remains unclear. Resolution of fatty livers in obese mice treated with Gly-MCA may also be independent of the obese phenotype since HFD-induced obese mice or genetically obese *db/db* mice displayed reduced steatosis with Gly-MCA, although these mice, even with weight loss, were still obese. Hepatic steatosis is positively correlated with hypertriglyceridemia. Surprisingly, Gly-MCA improved HFD-induced hepatic triglyceride contents but not serum triglyceride levels. The downregulation of hepatic lipogenesis-related mRNAs *Srebp1c*, *Cidea*, *Fasn* and *Elovl6* mainly led to decreased HFD-induced hepatic triglyceride accumulation after Gly-MCA treatment. It is well known that hepatic triglyceride secretion plays an important role in maintenance of serum triglyceride homoeostasis. Gly-MCA treatment did not affect the expression of hepatic triglyceride secretion-related genes such as *Mttp* and *Apob* (C.X. and C.J., unpublished observations). This result might partially explain the difference between liver and serum triglyceride levels after Gly-MCA treatment. Consistent with these findings, liver-specific CYP7A1 transgenic mice displayed reduced hepatic triglyceride levels and the unchanged serum triglyceride levels compared with WT mice^37^.

Ursodeoxycholic acid (UDCA), a bile acid metabolite, is used for the treatment of liver dysfunction, notably non-alcoholic fatty liver disease, non-alcoholic steatohepatitis and primary biliary cirrhosis^38^,^39^. Treatment with UDCA (28–35 mg kg^−1^ per day) for 1 year exerts beneficial metabolic profiles^40^. Three-week administration of UDCA at 20 mg kg^−1^ per day reduced serum AST, γ-glutamyl transferase, free fatty acids, total cholesterol and low-density lipoprotein cholesterol^41^. Hepatic gene expression changes and altered bile acid metabolites suggested that UDCA might be an inhibitor of FXR signalling; however, direct evidence that UDCA is an FXR antagonist was not presented. Others demonstrated that tauroursodeoxycholic acid (TUDCA), a UDCA derivative, exerts metabolic benefits as a chemical chaperone to inhibit endoplasmic reticulum (ER) stress in mice and human^42^,^43^. Interestingly, TUDCA antagonizes FXR activation by synthetic agonist GW4064, and TUDCA is rapidly hydrolyzed by gut bacterial BSH (C.X. and A.D.P., unpublished observations), suggesting that high doses of TUDCA might inhibit FXR signalling, resulting in improved insulin resistance.

A recent study revealed that the gut-restricted FXR agonist fexaramine, at much higher doses (100mgkg^−1^, administered by gavage) than those used for Gly-MCA (10mgkg^−1^, administered by pill), decreased diet-induced body weight gain and its associated metabolic dysfunction in the diet-induced obese mouse model^8^. However, in contrast to the present study, the effects of fexaramine appear to be driven in part by the activation of TGR5, whereas Gly-MCA works exclusively through the inhibition of intestinal FXR, with no effects on TGR5 signalling.

Emerging studies revealed that beige fat recruitment plays a significant role in whole-body energy metabolism^23^,^44^. Gly-MCA enhanced the browning of beige fat as a result of inhibition of the intestinal FXR-ceramide axis, and increased energy expenditure was evident even before significant weight loss was evident. Beige fat thermogenesis is sufficient to increase energy expenditure^34^. Supporting this view, activation of beige fat, but not classic brown fat, increased metabolic rates leading to decreased obesity^45^,^46^,^47^. Conversely, mice with selective inhibition of beige fat, displayed an increased susceptibility to HFD-induced body weight gain^24^. Other studies demonstrated that thermogenic adipose tissues from adult humans mainly express beige adipocyte-selective markers and not the classic brown adipocyte-related markers^34^,^48^. Genome-wide analyses based on global RNA sequencing further, indicated that beige adipocytes exist in supraclavicular regions of adult humans^49^. ^18^F-FDG-PET/CT analyses revealed that thermogenic adipocytes are undetectable under basal conditions, while prolonged cold treatment dramatically elicited thermogenic adipocytes accompanied by increased energy expenditure and improvement of insulin sensitivity^50^,^51^. Collectively, the existence of beige adipocytes in adult humans provides an attractive therapeutic target for obesity-related metabolic disease.

Lower intestinal-derived ceramides contributed to the metabolic benefits of Gly-MCA. In support of these findings, recent studies revealed that ceramides are correlated with obesity and insulin resistance^20^,^52^,^53^. Thus, it would be interesting to determine the effects of Gly-MCA on regulating body temperature in the cold. The expression of beige fat thermogenic-related genes were markedly induced by Gly-MCA treatment under thermoneutral, room temperature and cold conditions. Gly-MCA-treated mice displayed a higher core body temperature compared with vehicle-treated mice after cold stimulation (C.X. and C.J., unpublished observations). In addition, Gly-MCA had no effects on cAMP levels in both beige and brown fat. These results suggested that Gly-MCA treatment restored beige fat thermogenesis, likely independent of β-sympathetic nervous system activation. Thus, reduction of intestinal-derived ceramides largely mediated the Gly-MCA-increased energy expenditure. However, the mechanism by which ceramides compromise beige fat thermogenesis remains unclear. Fortuitously, ceramides are only partially reduced in mice treated with Gly-MCA and thus no toxicities would be expected nor are they observed in tissues dependent on ceramides such as neurons and skin. However, long-term studies are warranted to determine safety during chronic treatment.

Oral administration of a new chemical entity Gly-MCA prevents and reverses obesity-related metabolic dysfunctions. It is proposed that any compound that is orally administered, specifically inhibits intestinal FXR and has no effects on liver FXR signalling, would have utility in the therapy of patients with metabolic disorders.

## Methods

### Materials and reagents

Bile acids were ordered from Steraloids, Inc., (Newport, RI) and Sigma-Aldrich (St Louis, MO), and T-β-MCA-d5 sodium salt was from Toronto Research Chemicals, Inc., (Toronto, Ontario). C16:0, C18:0, C20:0, C22:0, C24:0 and C24:1 ceramides were obtained from Avanti Polar Lipids. HFD (60 kcal % fat) were purchased from Bio-Serv (Frenchtown, NJ). Gly-MCA was synthesized as according to the Supplementary Fig. 16.

### Subjects

The biopsies of the distal ileum mucosa tissue were obtained from 66 individuals who underwent colonoscopy. The ages of the subjects ranged from 18 to 67 years, and all had a BMI between 17.9 and 32.1 kg m^−2^. In these subjects, lean subjects (*n*=36), BMI are under 25 kg m^−2^, obese subjects (*n*=30), BMI are equal or over 25 kg m^−2^. The demographic characteristics were similar at baseline between the groups (Supplementary Table 2). All individuals fulfilled the following inclusion criteria: (1) no significant alcohol consumption (the definition of significant alcohol consumption has been inconsistent and ranged from >1 alcoholic beverage (10 g of alcohol per one drink unit) per day to >40 g per day); (2) no significant acute or chronic inflammatory disease; (3) no medical history of hypertension (that is, systolic blood pressure <140 mm Hg and diastolic blood pressure <85 mm Hg); (4) no clinical evidence of either cardiovascular or peripheral artery disease; (5) no thyroid dysfunction; (6) no gastrointestinal disease; and (7) no pregnancy. The study was approved by Conjoint Health Research Ethics Board of Peking University People's Hospital, and written informed consents were given all subjects before participation in this study.

### Animal studies

HFD (60% kcal from fat) was purchased from Bio-Serv, Inc. Male 6- to 8-week-old intestine-specific *Fxr*-null (*Fxr*^ΔIE^)^19^ mice and control (*Fxr*^fl/fl^) mice were on a C57BL/6N genetic background, after backcrossing with C57BL/6N mice for over 10 generations. For the Gly-MCA study, Gly-MCA was custom synthesized. For oral administration of Gly-MCA (0.25 mg Gly-MCA per pill, dose of 10 mg kg^−1^), bacon-flavoured dough pills were produced by adding Gly-MCA to the dough and then formulating the pills by use of a tablet triturate mold (Gallipot, St. Paul, MN)^54^. Mice were trained to eat the dough pills before the study. For the prevention of obesity, insulin resistance and hepatic steatosis, male WT C57BL/6 N mice, 6 to 8 weeks old, were fed a HFD (Bio-Serv, Frenchtown, NJ; 60 kcal% fat) and were orally administered with vehicle (control pills) or Gly-MCA (0.25 mg per pill per day, dose10 mg kg^−1^). For the therapy of obesity, obese C57BL/6N mice, induced by a HFD for 12 weeks, were administered (0.25 mg Gly-MCA per pill, dose of 10 mg kg^−1^). Leptin receptor-deficient *db/db* mice, 6 to 8 weeks old, fed a chow diet, were administered Gly-MCA (0.25 mg per pill per day, or 10 mg kg^−1^ once per day). To investigate the role of intestine FXR in ceramide synthesis, 6- to 8-week-old male littermate *Fxr*^fl/fl^ and *Fxr*^ΔIE^ mice were fed a chow diet and administered TCA (400 mg kg^−1^, in saline) by gavage. To determine whether the action of Gly-MCA was FXR dependent, 6- to 8-week-old male *Fxr*^fl/fl^ and *Fxr*^ΔIE^ mice, were fed a HFD and orally administered vehicle (control pills) or Gly-MCA (0.25 mg per pill per day, dose 10 mg kg^−1^). For the GW4064 (10 mg kg^−1^) and ceramide turnover study, male 6-week-old C57BL/6N mice fed a HFD were divided into four groups: vehicle, Gly-MCA (0.25 mg per pill per day, dose 10 mg kg^−1^), GW4064+Gly-MCA (each pill contained 0.25 mg GW4064 and 0.25 mg Gly-MCA, each dose10 mg kg^−1^) and injected with ceramide (see below)+Gly-MCA (0.25 mg per pill per day, dose 10 mg kg^−1^) groups treated for 5 weeks. C16:0 ceramide (Avanti Polar Lipids) was dissolved in saline with 0.5% sodium carboxymethyl cellulose and 5% Tween 80. The mice were administered C16:0 ceramide at a dose of 10 mg kg^−1^ per day by intraperitoneal (i.p.) injection. Saline with 0.5% sodium carboxymethyl cellulose and 5% Tween 80 was injected to controls. For the short-time treatment, male 6-week-old C57BL/6N mice fed a HFD were orally administered with vehicle (control pills) or Gly-MCA (0.25 mg per pill per day, dose 10 mg kg^−1^) for 5 days. Mice were housed individually in their home cages. Cumulative food intake and TEE_bal_ were measured for 1 week for vehicle and Gly-MCA-treated mice fed a HFD for 6 to 7 weeks. The following equation was used to calculate TEE_bal_:


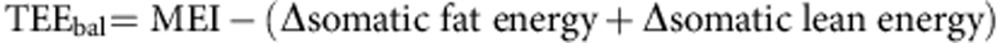


Metabolizable energy intake is defined as grams of food ingested per 24 h multiplied by the metabolizable energy for the HFD. ΔSomatic fat energy is calculated as final fat mass energy minus initial fat mass energy. ΔSomatic lean energy is calculated as final lean mass energy minus initial lean mass energy^55^. For thermal neutral temperature exposure experiments, male 6-week-old C57BL/6N mice fed a HFD were orally administered with vehicle (control pills) or Gly-MCA (0.25 mg per pill per day, dose10 mg kg^−1^) at 29.5 °C for 2 weeks. For cold stimulation experiments, male 6-week-old C57BL/6N mice fed a HFD were orally administered with vehicle (control pills) or Gly-MCA (0.25 mg per pill per day, dose 10 mg kg^−1^) at 22 °C for 5 days and then acutely moved to 5 °C for another 1 day. All mice were randomly assigned to experimental groups and the groups showed no difference in body weight gain before treatment. All mouse studies were performed in accordance with the Institute of Laboratory Animal Resources guidelines and approved by the NCI Animal Care and Use Committee.

### Indirect calorimetry

Indirect calorimetry was performed on obese mice fed a HFD for 12 weeks and then treated with vehicle or Gly-MCA (10 mg kg^−1^) concurrently on a HFD for 2 weeks using a 12-chamber Environment Controlled CLAMS (Columbus Instruments, Columbus, OH). After 48-h acclimatization, mice were monitored for 24 h at 22 °C and then for the following 24 h at thermoneutrality (29.5 °C) for recording data. During testing, food and water were provided *ad libitum* and Gly-MCA was administered via oral gavage.

### Metabolic assays

For the GTT, mice were fasted for 16 h, blood was drawn, and mice injected i.p. with 1 g kg^−1^ glucose. For the ITT, mice were fasted for 4 h, blood was drawn and then were injected with insulin (Eli Lilly, Washington, DC), i.p. at a dose of 1 U kg^−1^ body weight. Blood samples were taken from the tail at 15, 30, 60 and 90 min after injection, and glucose concentration was measured using a Glucometer (Bayer, Pittsburgh, PA).

### Histological analysis

Hematoxylin and eosin staining were performed on formalin-fixed paraffin-embedded sections using a standard protocol. At least three discontinuous liver sections were evaluated for each mouse.

### Triglycerides contents

Hepatic lipids were extracted using a 2:1 chloroform–methanol solution. Liver triglycerides were measured with a triglyceride colorimetric assay kit, according to the manufacturer's recommendation (Bioassay Systems, Hayward, CA).

### Cell culture

Caco-2 (ATCC HTB-37) cells were induced to differentiate using the following the method^56^: the cells were cultured in DMEM with 10% fetal bovine serum (FBS). The medium was changed once every 3 days. The cells were grown to 100% confluence, and then maintain 1 day. The cells were harvested with trypsin, diluted 1:3 and then seeded in a new plate, which was considered as one passage. After 50 passages, the differentiated Caco-2 cells were verified by mRNA expression of FXR and its target genes. The differentiated Caco-2 cells were incubated for 8 h with DMEM media with 1% FBS, and then exposed to Gly-MCA/CDCA/GW4064 for 24 h. HEK293T (ATCC CRL-3216) cells used for the luciferase report assays were obtained from the ATCC (Manassas, VA). The mycoplasma contamination tests on Caco-2 (ATCC HTB-37) and HEK293T (ATCC CRL-3216) were negative.

### Beige adipocytes

Stromal vascular cells (SVCs) were isolated from inguinal subcutaneous fat pads of normal male Sprague-Dawley rats (160–180 g). The fat pads were minced and digested in serum-free DMEM containing 0.8 mg ml^−1^ type I collagenase and 1% defatted BSA, for 60 min at 37 °C in a water bath shaken at 120 cycles per min. The digestion mixture was filtered through 80 and 400 steel mesh to remove debris and floating primary adipocytes. SVCs fractions containing adipose precursor cells residing in the digestion mixture were collected by centrifugation at 800 g for 10 min and plated in DMEM/F-12 (1:1) plus 10% FBS. Then, SVCs were differentiated into adipocytes in DMEM/F-12 (1:1) (10% FBS), supplemented with 5 μg ml^−1^ insulin, 33 μM biotin and 200 pM triiodothyronine. On differentiated day 2, the cells were treated with 1 μM rosiglitazone for another 5 days to differentiate into beige adipocyte. After 7 days of differentiation, cultured beige adipocytes were used for the next series of experiments^26^.

### Mitochondrial OCR assay

Primary SVCs were isolated from inguinal fat of s.d. rats. Subconfluent SVC cultures were trypsinized and seeded into 24-well plates (Seahorse XF24 extracellular flux analyser) and then differentiated into beige adipocytes. OCR of adipocytes in the basal state and in response to respiratory chain modulators were measured with XF Cell Mito Stress Test Kit (Seahorse Bioscience, North Billerica, MA) by use of a XF24 Flux Analyzer. The following modulators were injected sequentially: (1) Oligomycin (1 μM), (2) FCCP (1 μM), (c) Rotenone (1 μM) and Antimycin A (1 μM). OCR rates were calculated and analysed using the Seahorse XF24 v1.7.0.74 software.

### CCK-8 assay

Cell viability was determined using the Cell Counting Kit-8 assay (CCK-8, Dojindo Molecular Technologies, Gaithersburg, MD). A 100 μl aliquot of serum-free medium containing 10% CCK-8 was added to each well. The plates were incubated at 37 °C for 120 min, and absorbance measured at 450 nm. The absorbance of the treated cells was compared with that of the control cells exposed only to the vehicle, and thus were considered as 100% viable.

### Hydrolysis efficiency of Gly-MCA and T-β-MCA

Faecal proteins were prepared from faeces pellets (0.5 g) in pH 7.4 PBS (5.0 ml) using sonication. Incubations were carried out in 3 mM sodium acetate buffer, pH 5.2, containing 0.1 mg ml^−1^ faecal protein and 50 μM TβMCA-d5 or Gly-MCA in a final volume of 200 μl. After a 20 min incubation at 37 °C, the reactions were stopped by plunging the samples into dry ice. Acetonitrile (100 μl) was directly added to the reaction mix. After centrifuging at 14,000 *g* for 20 min, 5 μl of the supernatant was transferred to an auto sampler vial and subjected to analysis by use of a UPLC system coupled with a XEVO triple–quadrupole tandem mass spectrometer (Waters Corp., Milford, MA).

### Luciferase assays

Grace L. Guo supplied the PGL4-Shp-TK firefly luciferase construct and human FXR expression plasmid. Paul A. Dawson provided the human ASBT expression plasmid, and Makoto Miyazaki and Kristina Schoonjans gifted the pCMVSPORT6/hTGR5 and cAMP response element-driven luciferase reporter plasmids. The Cignal CRE Reporter Assay kit (LUC) was purchased from QIAGEN. HEK293T cells were co-transfected with: (1) a chimeric receptor construct in which the carboxy terminal portions of human FXR (containing the native ligand-binding domain and AF2 transactivation domain) was fused to an amino terminal GAL4 DNA-binding domain under regulatory control of the constitutively active SV40 promoter; (2) a firefly luciferase reporter plasmid driven by the UAS GAL4 DNA response element; and (3) a *Renilla* luciferase reporter gene (pRL-luciferase; Promega; Madison, WI) as a transfection efficiency control. The plasmids were transfected into cells using X-tremeGENE HP DNA Transfection Reagent (Roche). The cells were lysed, and luciferase activities measured with the Dual Luciferase Reporter Assay kit (Promega Corp., Madison, WI) and a Tecan GeniosPro luminescent plate reader (Research Triangle Park, NC).

### Quantification of faecal LCN-2

Frozen faecal samples were homogenized in PBS containing 0.1% Tween-20 (100 mg ml^−1^), vibrated for 20 min, and then centrifuged at 14,000 *g* for 10 min. Supernatants were collected and analysed with a mouse LCN-2 ELISA kit (R&D Systems, Minneapolis, MN).

### Determination of sphingomyelinase activity

Frozen intestine samples were homogenized in PBS containing 0.1% BSA, and then centrifuged at 18,000 *g* for 5 min. Supernatants were collected and analysed with a Colorimetric Sphingomyelinase Assay kit (Sigma-Aldrich).

### cAMP detection

Frozen intestine and subcutaneous adipose tissue samples were homogenized in 0.1 M HCl, and then centrifuged at 18,000 *g* for 5 min. Supernatants were collected and analysed with a mouse cAMP Direct Immunoassay kit (Abcam, Cambridge, MA).

### Assessment of serum cytokine and adipokine levels

Serum cytokine and adipokine levels were evaluated using the Milliplex MAP mouse cytokine/chemokine panel, rat/mouse hormone panel and mouse adiponectin panel on a Luminex (Bio-Plex 200) platform (Milliplex, Millipore, Luminex Corp., Austin, TX). Analysis was performed by Eve Technologies (Calgary, Alberta, Canada).

### RNA analysis

The intestine mucosa was gently scraped and liver were flash frozen in liquid nitrogen and were stored at −80 °C until RNA was prepared. RNA was extracted from frozen intestine and liver using TRIzol reagent (Invitrogen, Carlsbad, CA). Complementary DNA was synthesized from 1 μg total RNA using Superscript II reverse transcriptase (Invitrogen, Carlsbad, CA). Quantitative PCR primers were designed with qPrimerDepot, and the sequences are shown in the Supplementary Table 3. Messenger RNA levels were normalized to 18S ribosomal RNA and expressed as fold change relative to the control group.

### Western blotting

Intestine protein extracts were prepared, separated and transferred to a PVDF membrane. The membrane was incubated with antibody against SPTLC2 (LS-B9514, LifeSpan Biosciences, Inc., Seattle, WA) at 1:1000 dilution. Signals obtained were normalized to β*-*ACTIN (Abcam). For quantitation of the western blot bands, the blots were scanned and analysed using ImageJ software (National Institutes of Health, Bethesda, MD).

### Lipidomics analysis

For serum lipidomics, a 25 μl aliquot of serum was extracted with 100 μl of ice-cold chloroform:methanol (2:1, v/v) solution containing SM (17:0) and CER (17:0) (Avanti Polar Lipids, Alabaster, AL) at 2 μM as internal standards. The samples were vibrated for 30 s and then incubated for 5 min at room temperature. The organic and aqueous phases were separated by centrifugation at 13,000 *g* for 5 min. The lower organic phase was transferred to another clean tube and evaporated to dryness at room temperature under vacuum. The residue was dissolved in 25 μl of chloroform:methanol (1:1), followed by diluting with isopropanol:acetonitrile:H_2_O (2:1:1, v/v/v) containing 2 μM PC (17:0) before UPLC-MS analysis. For tissue lipidomics, about 50 mg tissue was accurately weighed and homogenized with 700 μl of methanol:H_2_O (4:3, v/v) solution and then extracted using 800 μl of chloroform containing SM (17:0) and CER (17:0) at 2 μM as internal standards. The homogenate was shaken and incubated at 37 °C for 20 min followed by centrifugation at 13,000 *g* for another 20 min. The lower organic phase was collected and evaporated to dryness under vacuum. The residue was then suspended with 100 μl of chloroform:methanol (1:1, v/v) solution and then diluted with isopropanol:acetonitrile:H_2_O (2:1:1, v/v/v) solution containing 2 μM PC (17:0) before injection. Lipidomics analysis was performed on an Acquity UPLC/Synapt QTOFMS system (Waters Corp., Milford, MA) equipped with an electrospray ionization (ESI) source. Separation was achieved on an Acquity UPLC CSH C18 column (100 × 2.1 mm internal diameter, 1.7 μm, Waters Corp.). The mobile phase was a mixture of acetonitrile/water (60/40, v/v, A) and isopropanol/acetonitrile (90/10, v/v, B), and both A and B contained 10 mM ammonium acetate and 0.1% formic acid. The gradient elution programme consisted of a 2 min linear gradient of 60% A to 57% A, to 50% A at 2.1 min*, a linear decrease to 46% A at 12 min, to 30% A at 12.1 min*, a linear decrease to 1% A at 18 min before returning to initial conditions at 18.5 min to equilibrate the column (*indicates ballistic gradient). The column temperature was maintained at 55 °C and the flow rate was 0.4 ml min^−1^. Mass spectrometry data were acquired in the both positive and negative ESI modes at a range of *m/z* 100–1,000.

Concentrations of bile acids were determined by an Acquity UPLC/Xevo G2 QTOFMS system (Waters Corp.) with an ESI source. An Acquity BEH C18 column (100 × 2.1 mm internal diameter, 1.7 μm, Waters Corp.) was applied for chromatographic separation. A mixture of 0.1% formic acid in water (A) and 0.1% formic acid in acetonitrile (B) was used as the mobile phase. The gradient elution was started from 80% A for 4 min, decreased linearly to 60% A over 11 min, to 40% A over the next 5 min, to 10% A for the succeeding 1 min, and finally increased to 80% A for 4 min to re-equilibrate the column. Column temperature was maintained at 45 °C, and the flow rate was 0.4 ml min^−1^. Mass spectrometry detection was operated in negative mode. A mass range of *m/z* 50–850 was acquired.

### Synthesis of Gly-MCA

Synthesis of β-MCA **9** and Gly-MCA **10**. β-MCA **9** was prepared as illustrated in the Supplementary Fig. 16 by following the literature procedure^57^. In general, esterification of the dihydroxy acid **1** with methanol under acid catalysis provides ester **2** in quantitative yield. Protection of the hydroxyl group in 3-position with ethyl chloroformate provided carbonate **3** in 79% yield. Oxidation of the 6-hydroxyl group with potassium chromate gave ketone **4** in quantitative yield. Bromination with 47% HBr solution gave bromo ketone **5**, which upon reduction with NaBH_4_ gave bromohydrin **6**, in 73% yield. Reductive dehydrobromination with zinc metal provides olefin **7** in about 80% yield. Cis-dihydroxylation with osmium tetroxide and *N*-methymorpholine *N*-oxide gave cis diol **8** in moderate yield followed by hydrolysis provided β-MCA **9** in quantitative yield. β-MCA **9** was conjugated to provide Gly-MCA **10** as shown in Supplementary Fig. 16. In brief, ethyl glycinate hydrochloride (1 mmol) was suspended in ethyl acetate (20 ml) containing triethylamine (0.3 ml). The reaction mixture was stirred at 25 °C for 1 h and reacted with β-MCA **9** (0.70 mmol) and EEDQ (1 mmol) by refluxing overnight. The resultant reaction mixture was poured over water (10 ml) and ethyl acetate (10 ml). The organic layer was separated, washed successively with 0.5 N NaOH (10 ml), water (10 ml), 0.5 N HCl (10 ml × 2), water (10 ml × 2), dried over Drierite, filtered and evaporated. The residue obtained was purified by column chromatography and methyl Gly-β-muricholic obtained was dissolved in boiling ethanol (15 ml) and hydrolyzed with 10% K_2_CO_3_ over a period of 1 h. The solvent was evaporated to half its volume, diluted with water (10 ml) and the aqueous solution was acidified to give Gly-MCA **10** as a white powder in 61% yield over 2 steps. ^1^H NMR spectra were recorded on a Bruker Avance II 500 MHz instrument equipped with a BBO probe and the chemical shifts are given as *δ* values with reference to tetramethylsilane as internal standard. melting point 224–226 °C; ^1^H NMR (500 MHz, CD_3_OD) 0.75 (s, 3H, 18-Me), 1.01 (d, 3H, J=6.5 Hz, 21-Me), 1.12 (s, 3H, 19-Me), 1.05–1.56 (m, 13H), 1.58–1.70 (m, 2H), 1.71–1.80 (m, 2H), 1.80–2.03 (m, 3H), 2.04–2.10 (m, 1H), 2.15–2.25 (m, 1H), 2.29–2.37 (m, 1H), 3.30–3.35 (m, 1H), 3.44–3.56 (m, 2H), 3.58–3.61 (m, 1H), 3.70–3.73 (m, 2H), 3.91 (s, 2H); 13C-NMR (125 MHz, CD_3_OD): d 175.75, 171.84, 75.81, 72.98, 70.47, 55.89, 55.17, 47.79, 43.43, 40.56, 40.06, 39.79, 38.17, 35.42, 35.37, 35.15, 33.57, 32.46, 31.74, 29.34, 28.22, 26.83, 24.78, 20.64, 17.69, 11.27; [M+H]+ calculated for C_26_H_43_NO_6_, 466.3169; found 466.3133; [M−H]- calculated for C_26_H_43_NO_6_, 464.3012, found 464.3055. Purity was determined to be >97%.

### Data analysis

Sample sizes were chosen based on pilot experiments that ensured adequate statistical power with similar variances. Values that fell more than two s.d. from the mean were considered as outliers and were excluded from statistical analyses. Experimental values are presented as mean±s.d. Appropriate statistical analyses were applied, assuming a normal sample distribution. When comparing two groups, statistical significance was determined using two-tailed Student's *t*-test. When more than two groups were investigated, one-way analysis of variance with Tukey's correction was applied for comparisons between different preservation groups. *P* values of <0.05 were considered significant. The investigators involved in this study were not completely blinded during sample collection or data analysis. All data shown were representative results from at least three independent experiments.

## Additional information

**How to cite this article:** Jiang, C. *et al.* Intestine-selective farnesoid X receptor inhibition improves obesity-related metabolic dysfunction. *Nat. Commun.* 6:10166 doi: 10.1038/ncomms10166 (2015).

## Supplementary Material

Supplementary Information Supplementary Figures 1-17, Supplementary Tables 1-3 and Supplementary Reference
