# Supplementary material for: Intestine-selective farnesoid X receptor inhibition improves obesity-related metabolic dysfunction
Source: Nat Commun. 2015 Dec 15;6:10166. doi: 10.1038/ncomms10166 (PMC4682112; doi:10.1038/ncomms10166)
Supplement: Supplementary Information — Supplementary Figures 1-17, Supplementary Tables 1-3 and Supplementary Reference [file ncomms10166-s1.pdf]

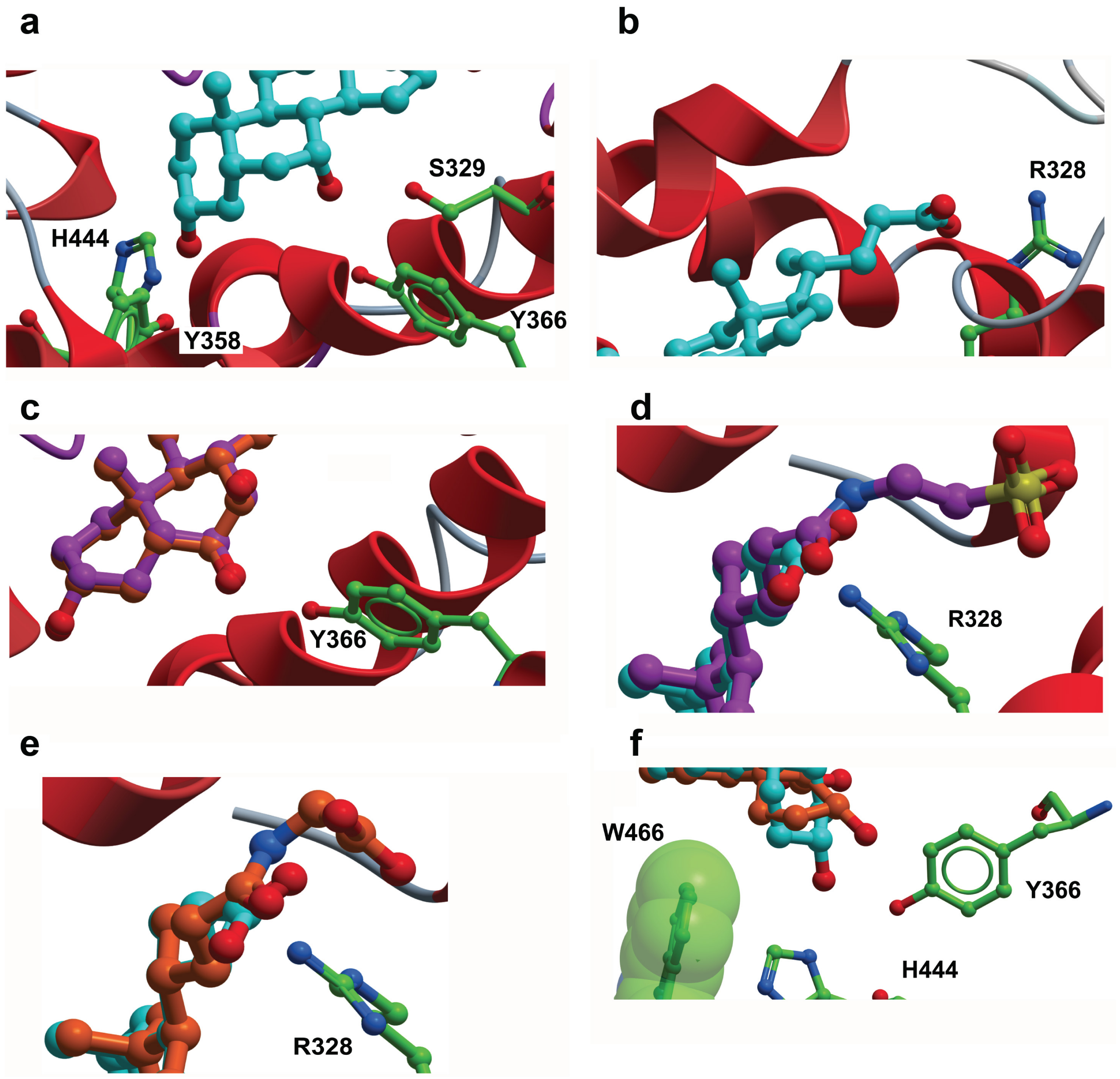

Supplementary Figure 1. Ligand docking of FXR agonist and antagonist.
Docking of agonist CDCA (a, b, d, e, f), antagonist T-β-MCA (c, d) and Gly-MCA (c, e, f) into the human FXR-LBD in the agonist conformation (Molsoft ICM). CDCA, T-β-MCA and Gly-MCA are displayed as sticks and colored by atom type, with carbon atoms in cyan (CDCA), magenta (T-β-MCA) and orange (Gly-MCA); protein residues are displayed as stick with the carbon atoms colored in green.
(f) Superimposition of the bent shape A/B junction cis S1 (CDCA) and S2 (Gly-MCA). Secondary structure is displayed as ribbon. Protein-ligand hydrogen bond and salt bridge interactions are displayed as dashed black and red lines, respectively (Molsoft ICM).

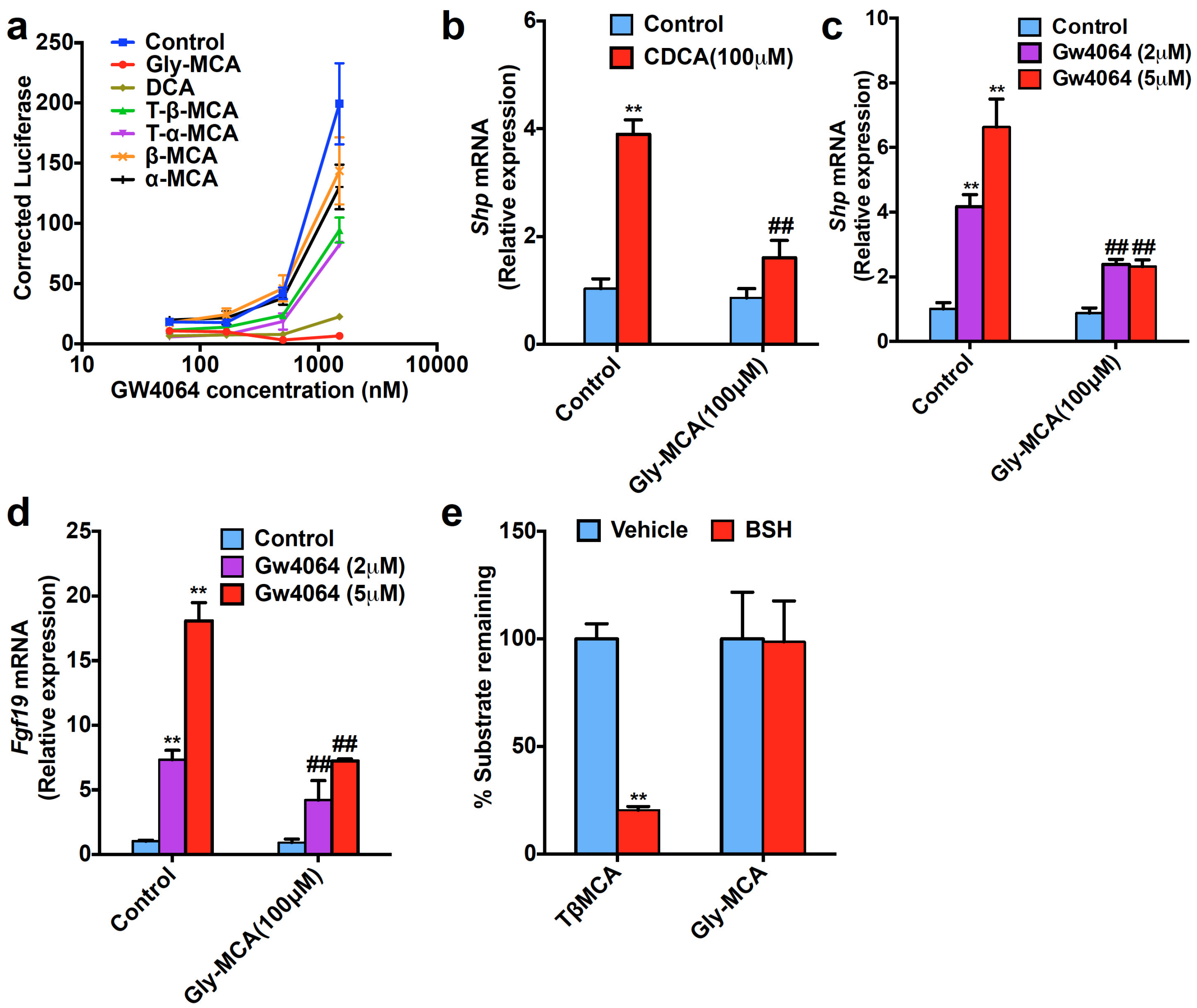

Supplementary Figure 2. Gly-MCA is a FXR antagonist of both human and mouse.
(a) Luciferase activity in HEK293T fibroblasts transiently co-transfected with a PGL4-Shp-TK firefly luciferase construct, the control plasmid phRL-SV40, human FXR and human ASBT expression plasmids as a function of concentration of the added FXR agonist GW4064 in the presence of control, 50 μM of either Gly-MCA, DCA, T-β-MCA, T-α-MCA, β-MCA, or α-MCA. Data are presented as mean ± sd.
(b, c) *Shp* mRNA expression of differentiated Caco2 cells after treatment with 100 μM Gly-MCA with 100μM CDCA (b), 2μM, or 5μM GW4064 (c) ($n = 3$). Data are presented as mean ± sd. Expression was normalized to 18S RNA. One-way ANOVA with Tukey's correction. $^{**}P < 0.01$ compared to control, $^{\#\#}P < 0.01$ compared to CDCA (b) or GW4064 (c) treatment.
(d) Levels of *Fgf19* mRNAs in differentiated Caco2 cells after treatment with 100 μM Gly-MCA with 2μM, 5μM GW4064 ($n = 3$). Data are presented as mean ± sd. Expression was normalized to 18S RNA. One-way ANOVA with Tukey's correction. $^{**}P < 0.01$ compared to control, $^{\#\#}P < 0.01$ compared to GW4064 treatment.
(e) Bile acids hydrolysis efficiency detected by BSH ($n = 3$). Data are presented as mean ± sd. Two-tailed Student's t-test. $^{**}P < 0.01$ compared to vehicle.

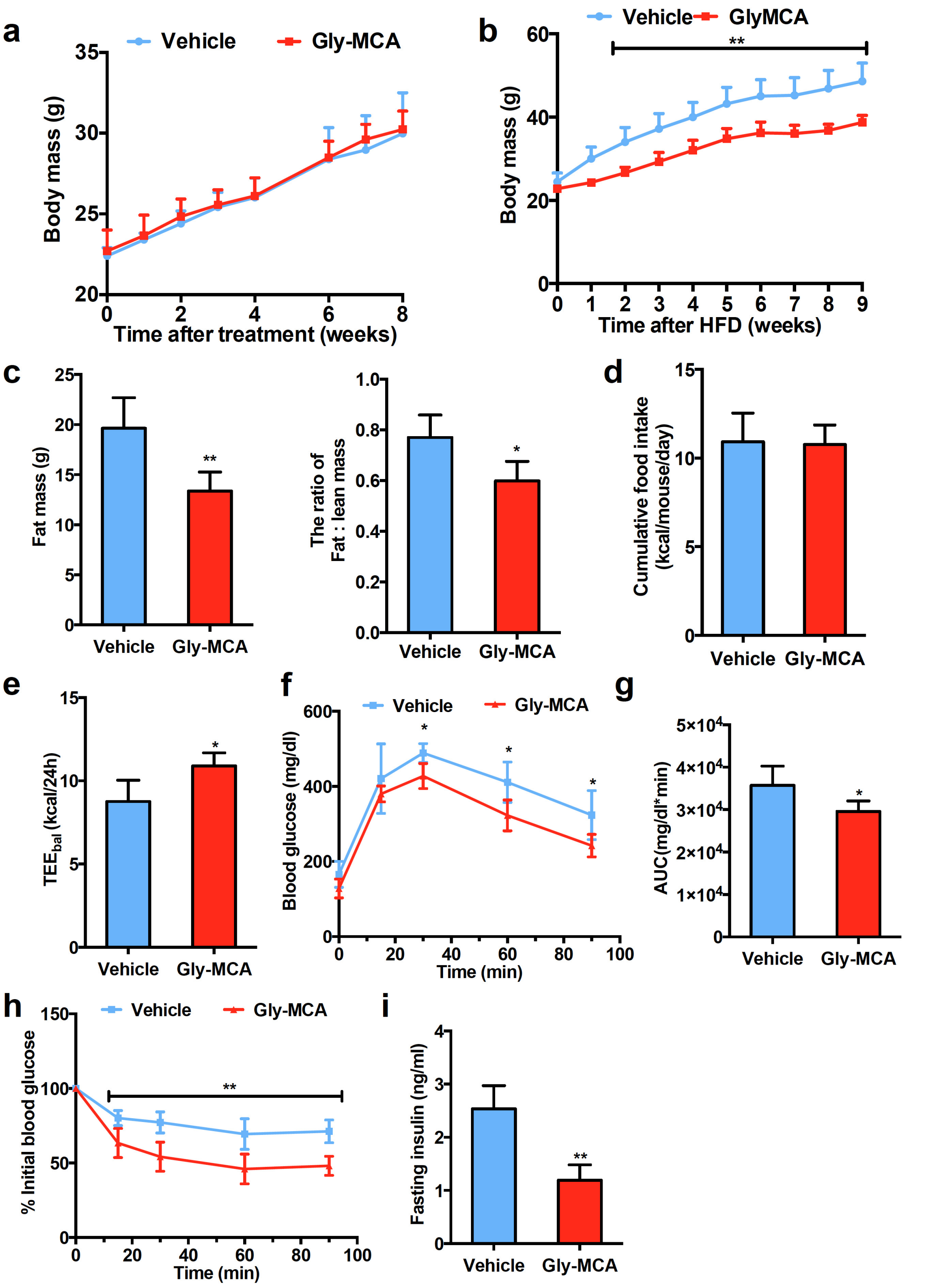

Supplementary Figure 3. Gly-MCA treatment protects mice from HFD-induced obesity and insulin resistance.

(a) The growth curves of body weight of vehicle- and Gly-MCA-treated mice fed a chow diet. $n = 5$ mice per group. Data are presented as mean ± sd. Two-tailed Student's t-test.

(b) The growth curves of body weight of vehicle- and Gly-MCA-treated mice fed a HFD. $n = 5$ mice per group. Data are presented as mean ± sd. Two-tailed Student's t-test. $^{**}P < 0.01$ compared to vehicle-treated mice.
(c) Body composition as determined by NMR to show the fat mass (left panel) and fat mass to lean mass ratio (right panel) in vehicle and Gly-MCA-treated mice, respectively, after 9 weeks on a HFD. $n = 5$ mice per group. Data are presented as mean ± sd. Two-tailed Student's t-test. $^{*}P < 0.05$, $^{**}P < 0.01$ compared to vehicle-treated mice.
(d) Cumulative food intake per day averaged over a period of one week (from 6 to 7 weeks) in vehicle- and Gly-MCA-treated mice fed a HFD. $n = 5$ mice per group. Data are presented as mean ± sd. Two-tailed Student's t-test.
(e) Energy expenditure using an indirect energy balance (TEE_{bal}) for an average period of one week (from 6 to 7 weeks) in vehicle- and Gly-MCA-treated mice fed a HFD. $n = 5$ mice per group. Data are presented as mean ± sd. Two-tailed Student's t-test. $^{*}P < 0.05$ compared to vehicle-treated mice.
(f, g) Glucose tolerance test (f) and the area under the curve (AUC) (g) in vehicle- and Gly-MCA-treated mice after 4 weeks on a HFD. $n = 5$ mice per group. Data are presented as mean ± sd. Two-tailed Student's t-test. $^{*}P < 0.05$ compared to vehicle-treated mice.
(h) Insulin tolerance test (ITT) in vehicle- and Gly-MCA-treated mice fed a HFD for 5 weeks. $n = 5$ mice per group. Data are presented as mean ± sd. Two-tailed Student's t-test. $^{**}P < 0.01$ compared to vehicle-treated mice.
(i) Fasting serum insulin levels of vehicle- and Gly-MCA-treated mice fed a HFD for 9 weeks. $n = 5$ mice per group. Data are presented as mean ± sd. Two-tailed Student's t-test. $^{**}P < 0.01$ compared to vehicle-treated mice.

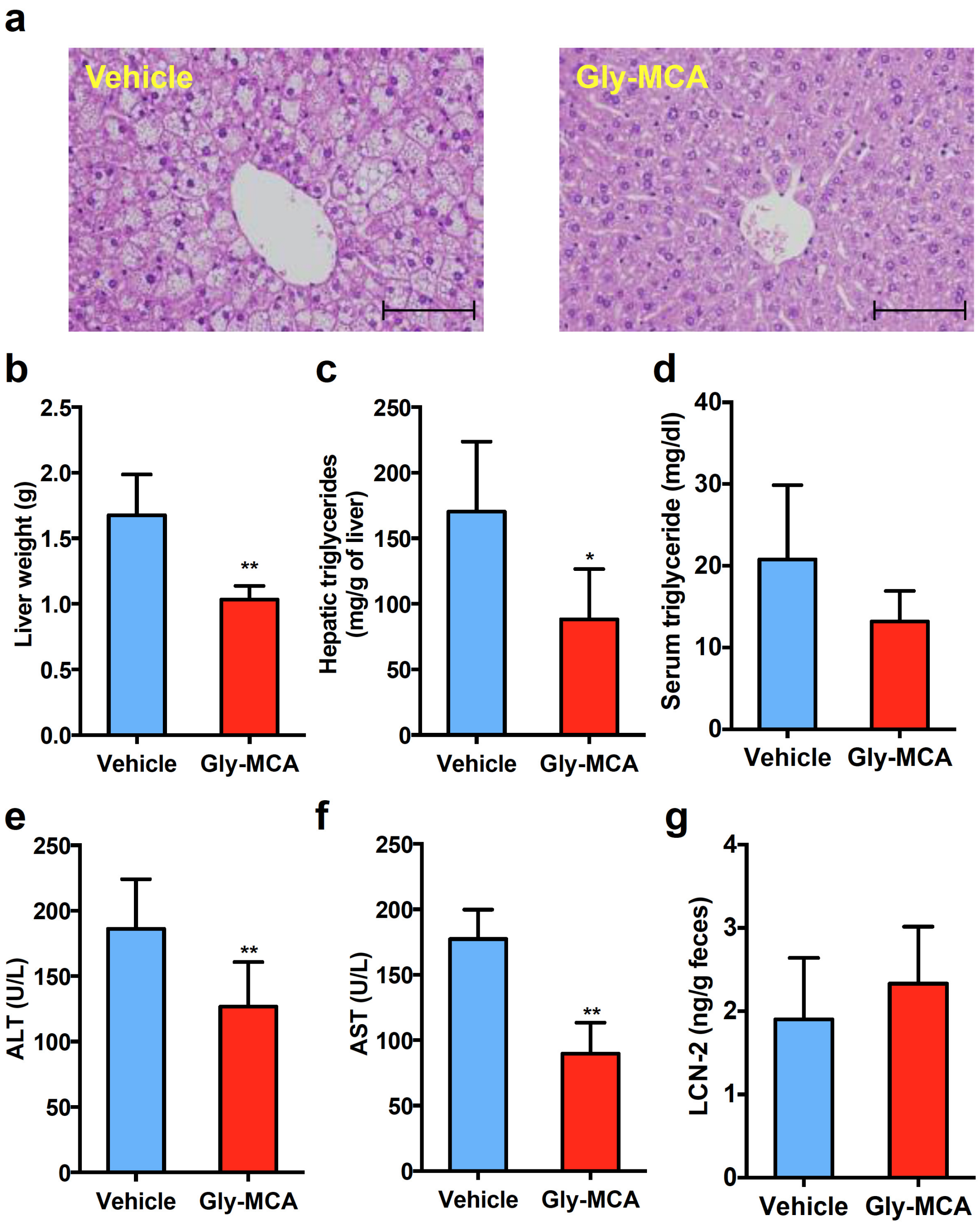

Supplementary Figure 4. Gly-MCA decreases HFD-induced hepatic steatosis.
The mice fed a HFD were treated concurrently with or without Gly-MCA for 9 weeks.
(a) Representative H&E staining of liver sections. $n = 5$ mice per group.
(b) Liver weights. $n = 5$ mice per group. Data are presented as mean ± sd. Two-tailed Student's t-test. $^{**}P < 0.01$ compared to vehicle-treated mice.
(c, d) Liver (c) and serum (d) triglyceride contents. $n = 5$ mice per group. Data are presented as mean ± sd. Two-tailed Student's t-test. $^{*}P < 0.05$ compared to vehicle-treated mice.
(e, f) Serum ALT (e) and AST (f) levels. $n = 5$ mice per group. Data are presented as mean ± sd. Two-tailed Student's t-test. $^{**}P < 0.01$ compared to vehicle-treated mice.
(g) Fecal LCN-2 concentrations. $n = 5$ mice per group. Data are presented as mean ± sd. Two-tailed Student's t-test.

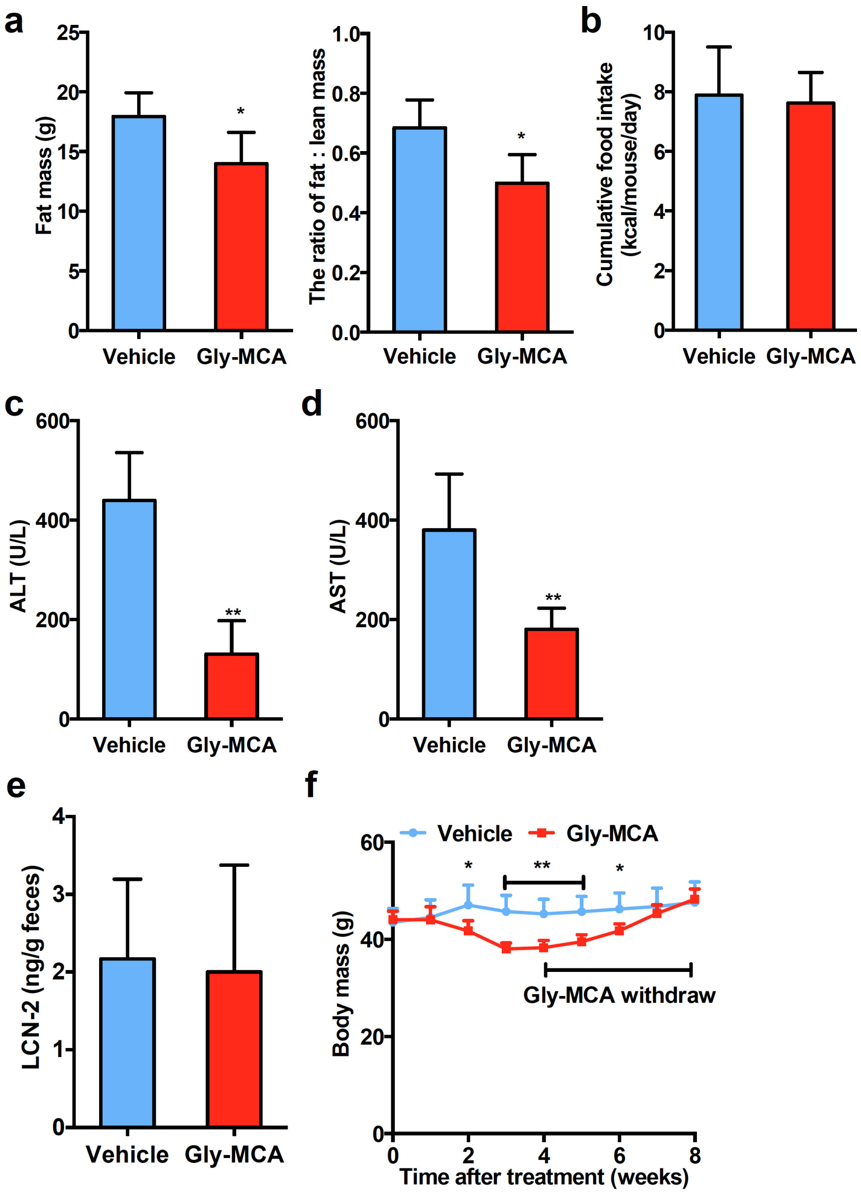

Supplementary Figure 5. Gly-MCA treatment has no effect on food intake, liver and intestine function of obese mice fed a HFD.

(a) Fat mass (left panel) and fat mass to lean mass ratio (right panel) of HFD-induced obese mice after 2 weeks of Gly-MCA treatment. n=5 mice per group. n = 5 mice per group. Data are presented as mean ± sd. Two-tailed Student's t-test. *P < 0.05 compared to vehicle-treated mice.

(b) Cumulative food intake per day averaged over a period of one week (from 2 to 3 weeks) in vehicle- and Gly-MCA-treated obese mice fed a HFD. n = 5 mice per group. Data are presented as mean ± sd. Two-tailed Student's t-test.

(c, d) Serum ALT (c) and AST (d) levels in obese mice after 5 weeks of Gly-MCA treatment. n = 5 mice per group. Data are presented as mean ± sd. Two-tailed Student's t-test. **P < 0.01 compared to vehicle-treated mice.

(e) Fecal LCN-2 concentrations in obese mice after 5 weeks of Gly-MCA treatment. n = 5 mice per group. Data are presented as mean ± sd. Two-tailed Student's t-test.

(f) Body weight of mice treated with or without Gly-MCA, and Gly-MCA treatment removal after 4 weeks. n = 5 mice per group. Data are presented as mean ± sd. Two-tailed Student's t-test. *P < 0.05, **P < 0.01 compared to vehicle-treated mice.

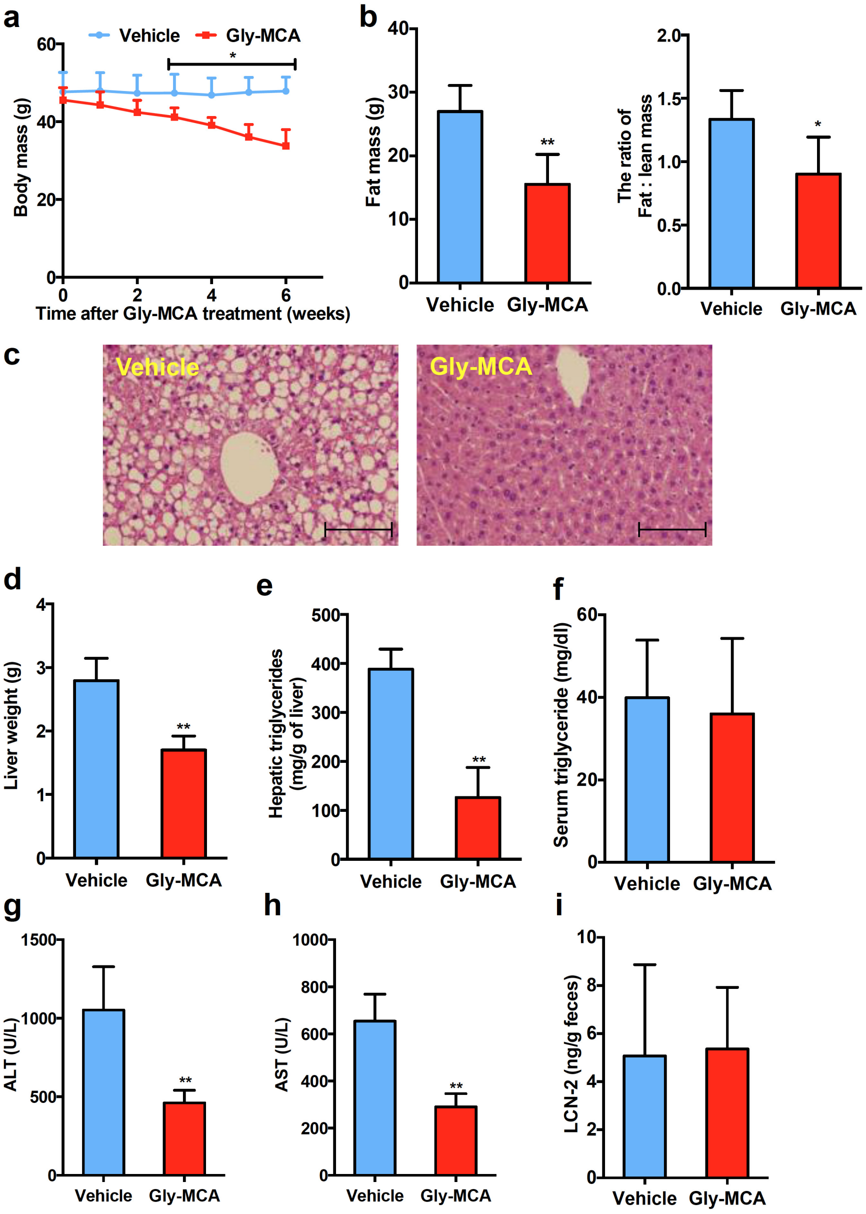

Supplementary Figure 6. Gly-MCA reverses obesity related metabolic disorders in *db/db* mice.
db/db mice were treated with or without Gly-MCA for 6 weeks.
(a) The growth curves of body weight. n = 5 mice per group. Data are presented as mean ± sd. Two-tailed Student's t-test. *P < 0.05 compared to vehicle treatment.
(b) Fat mass (left panel) and fat mass to lean mass ratio (right panel). n = 5 mice per group. Data are presented as mean ± sd. Two-tailed Student's t-test. *P < 0.05, **P < 0.01 compared to vehicle treatment.
(c) Representative H&E staining of liver sections. n = 5 mice per group.
(d) Liver weights. n = 5 mice per group. Data are presented as mean ± sd. Two-tailed Student's t-test. **P < 0.01 compared to vehicle treatment.
(e, f) Liver (e) and serum (f) triglyceride contents. n = 5 mice per group. Data are presented as mean ± sd. Two-tailed Student's t-test. **P < 0.01 compared to vehicle treatment.
(g, h) Serum ALT (g) and AST (h) levels. n = 5 mice per group. Data are presented as mean ± sd. Two-tailed Student's t-test. **P < 0.01 compared to vehicle treatment.
(i) LCN-2 levels in feces. n = 5 mice per group. Data are presented as mean ± sd. Two-tailed Student's t-test.

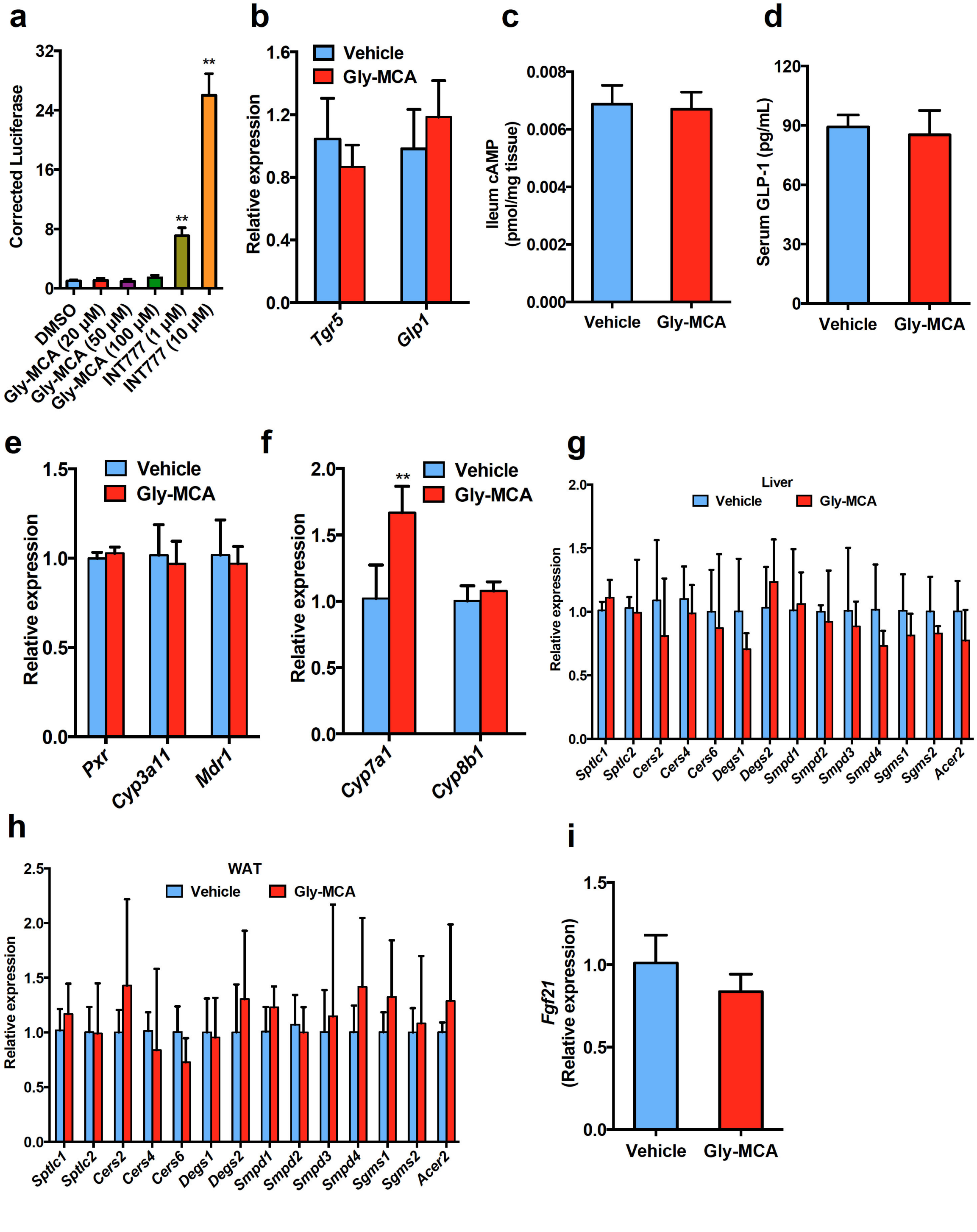

Supplementary Figure 7. Gly-MCA selectively inhibits intestinal FXR signaling.

(a) Luciferase assays for TGR5 activation. HEK293 cells were transfected with TGR5 expression vector and cAMP-response element luciferase in the presence of vehicle and indicated concentrations of Gly-MCA or INT-777 as a positive control. Six replicates of three separate experiments. Data are presented as mean ± sd. Two-tailed Student's t-test. $^{**}P < 0.01$ compared to control (DMSO).

(b) mRNA levels for *Tgr5* and *Glp1* in ileum. Mice fed a HFD were treated concurrently for 9 weeks with or without Gly-MCA. Expression was normalized to 18S RNA. $n = 5$ mice per group. Data are presented as mean ± sd. Two-tailed Student's t-test.

(c) Ileum cAMP levels. Mice fed a HFD were treated concurrently for 9 weeks with or without Gly-MCA.. n = 5 mice per group. Data are presented as mean ± sd. Two-tailed Student's t-test.
(d) Serum GLP-1 levels. Mice fed a HFD were treated concurrently for 9 weeks with or without Gly-MCA.. n = 5 mice per group. Data are presented as mean ± sd. Two-tailed Student's t-test.
(e) mRNA levels for *Pxr* target genes in ileum. Mice fed a HFD were treated concurrently for 9 weeks with or without Gly-MCA.. Expression was normalized to 18S RNA. n = 5 mice per group. Data are presented as mean ± sd. Two-tailed Student's t-test.
(f) mRNA levels for *Cyp7a1* and *Cyp8b1* in liver. Mice fed a HFD were treated concurrently for 9 weeks with or without Gly-MCA.. Expression was normalized to 18S RNA. n = 5 mice per group. Data are presented as mean ± sd. Two-tailed Student's t-test. **$P < 0.01$ compared to vehicle-treated mice.
(g, h) The expressions of mRNAs encoded by ceramide synthesis- and catabolism- related genes in liver (g) and WAT (h). Mice fed a HFD were treated concurrently for 9 weeks with or without Gly-MCA. Expression was normalized to 18S RNA. n = 5 mice per group. Data are presented as mean ± sd. Two-tailed Student's t-test.
(i) mRNA levels for *Fgf21* in liver. The mice were treated with or without Gly-MCA for 9 weeks concurrently on a HFD. Expression was normalized to 18S RNA. n = 5 mice per group. Data are presented as mean ± sd. Two-tailed Student's t-test.

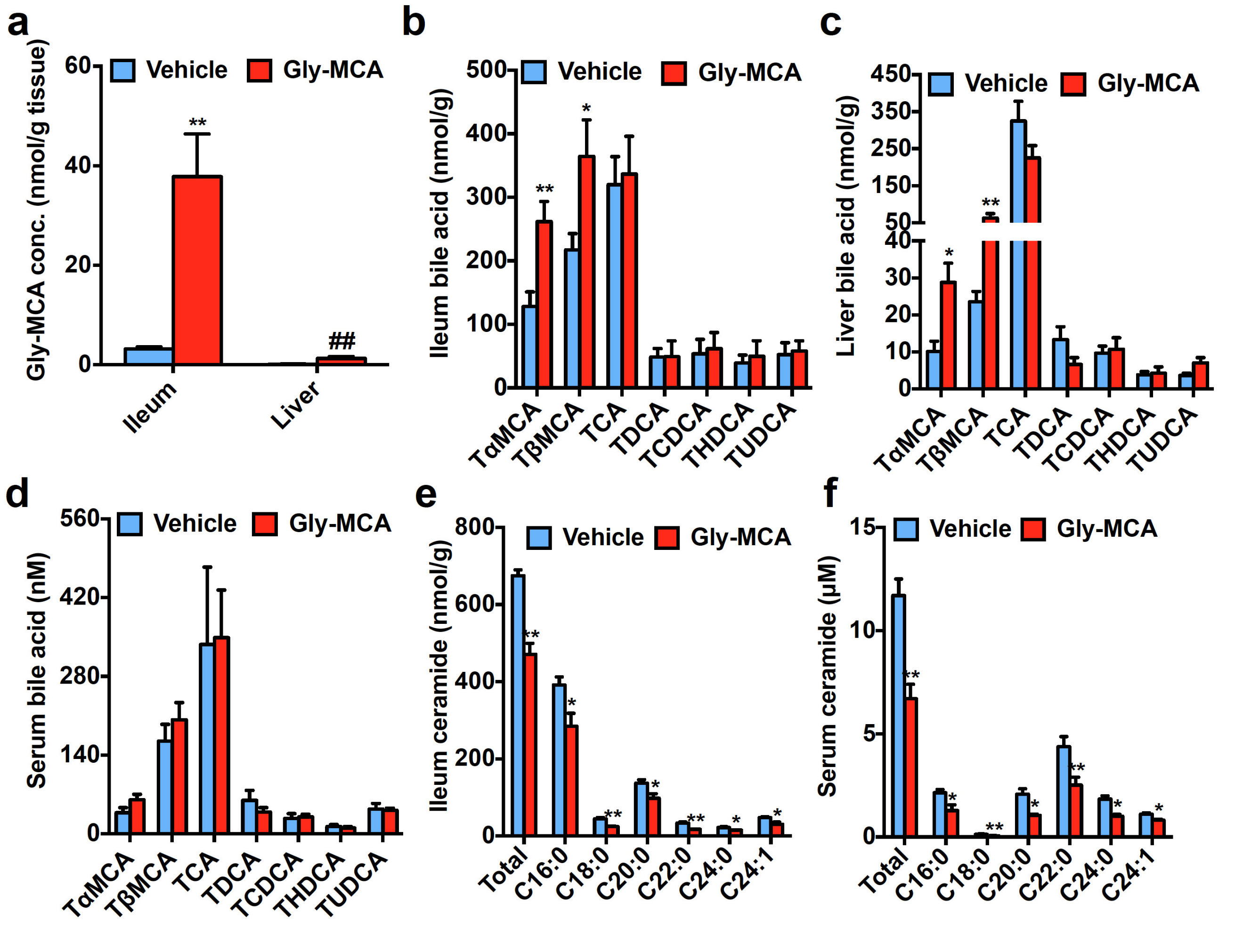

Supplementary Figure 8. Gly-MCA regulates bile acid composition and ceramide metabolism of obese mice fed a HFD.

Mice fed a HFD for 5 weeks were treated concurrently with or without Gly-MCA.

(a) Gly-MCA levels in ileum and liver. $n = 5$ mice per group. Data are presented as mean ± sd. One-way ANOVA with Tukey's correction. ***P* < 0.01 compared to vehicle treatment. ##*P* < 0.01 compared to Gly-MCA levels of ileum after Gly-MCA treatment.

(b-d) Individual taurine-conjugated bile acids levels in ileum (b), liver (c) and serum (d). $n = 5$ mice per group. Data are presented as mean ± sd. Two-tailed Student's t-test. **P* < 0.05, ***P* < 0.01 compared to vehicle treatment.

(e, f) Ileum (e) and serum (f) ceramides levels. $n = 5$ mice per group. Data are presented as mean ± sd. Two-tailed Student's t-test. **P* < 0.05, ***P* < 0.01 compared to vehicle treatment.

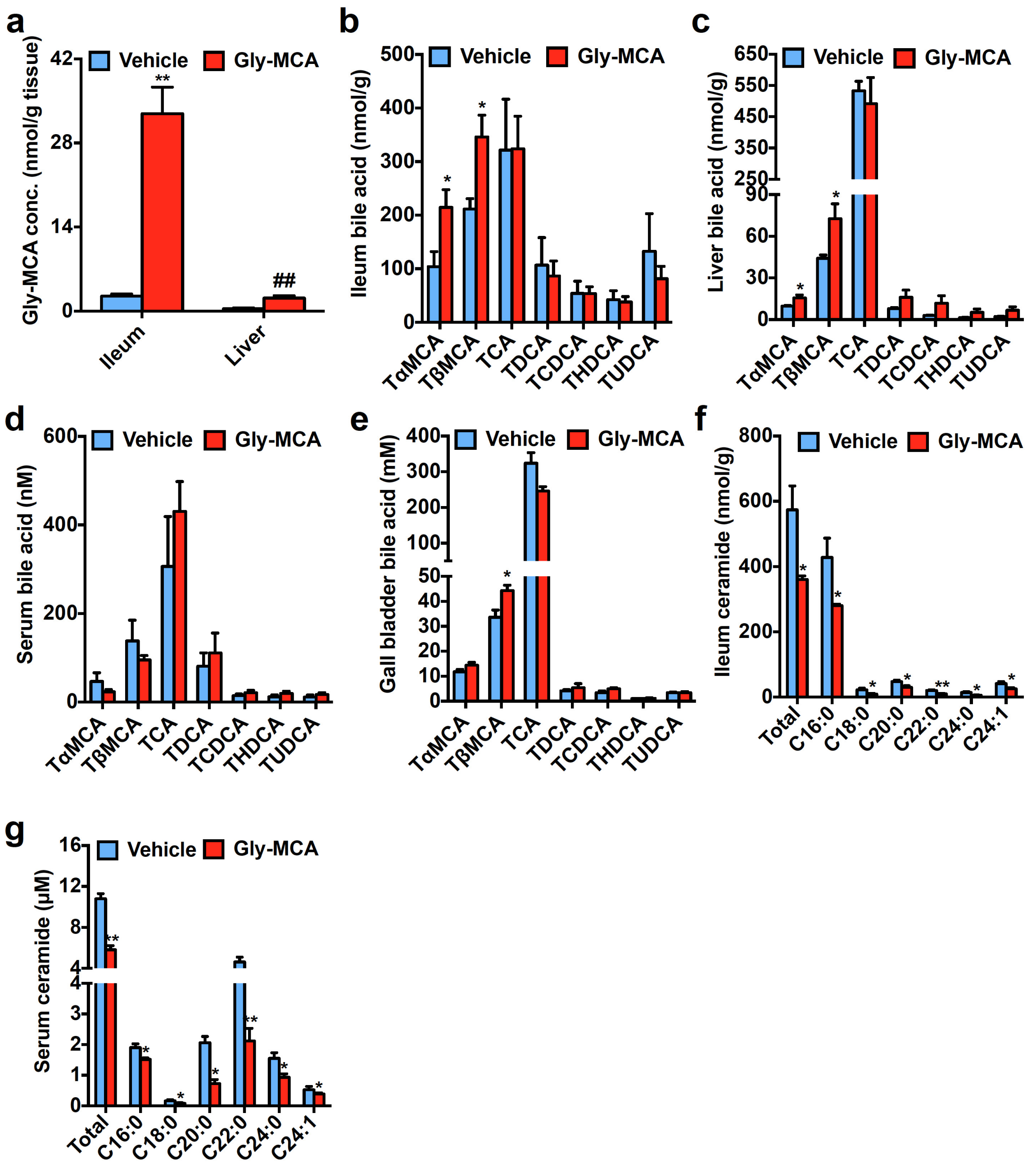

Supplementary Figure 9. Gly-MCA regulates bile acid composition and ceramide metabolism of *db/db* mice. *db/db* mice were treated with or without Gly-MCA for 6 weeks.

(a) Gly-MCA levels in ileum, and liver. n = 5 mice per group. Data are presented as mean ± sd. One-way ANOVA with Tukey's correction. **P < 0.01 compared to vehicle treatment. ##P < 0.01 compared to Gly-MCA levels of ileum after Gly-MCA treatment.

(b-e) Individual taurine-conjugated bile acids levels in ileum (b), liver (c), serum (d) and gall bladder (e). n = 5 mice per group. Data are presented as mean ± sd. Two-tailed Student's t-test. *P < 0.05, **P < 0.01 compared to vehicle treatment.

(f, g) Ileum (f) and serum (g) ceramides levels. n = 5 mice per group. Data are presented as mean ± sd. Two-tailed Student's t-test. *P < 0.05, **P < 0.01 compared to vehicle treatment.

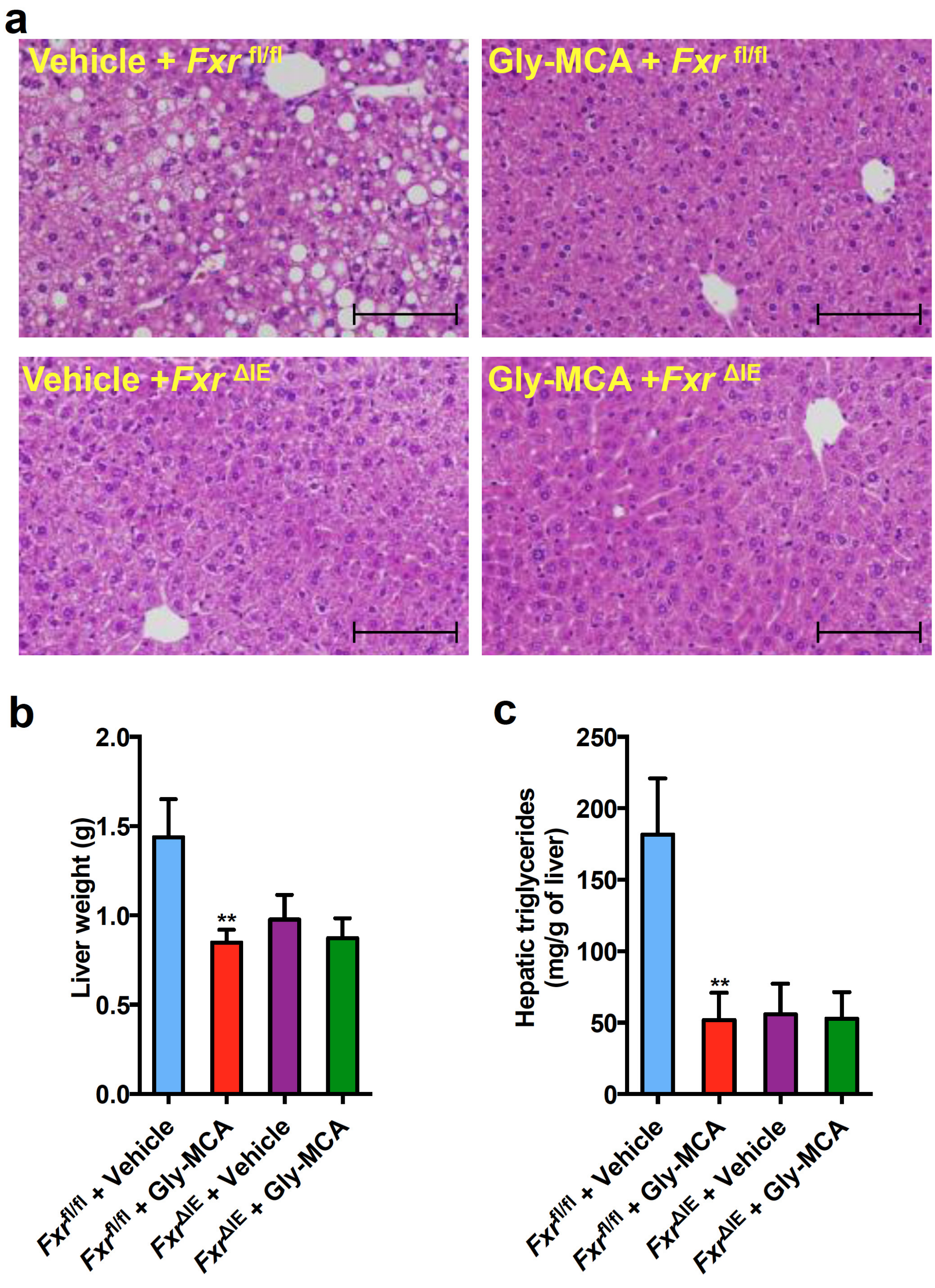

Supplementary Figure 10. Gly-MCA reduces hepatic steatosis via the inhibition of intestinal FXR.
Fxr$^{fl/fl}$ mice and *Fxr*$^{\Delta IE}$ mice fed a HFD 8 weeks were treated concurrently with vehicle or Gly-MCA.
(a) Representative H&E staining of liver sections. n = 5 mice per group.
(b) Liver weights. n = 5 mice per group. Data are presented as mean ± sd. One-way ANOVA with Tukey's correction. $^{**}P < 0.01$ compared to vehicle-treated *Fxr*$^{fl/fl}$ mice.
(c) Liver triglyceride contents. n = 5 mice per group. Data are presented as mean ± sd. One-way ANOVA with Tukey's correction. $^{**}P < 0.01$ compared to vehicle-treated *Fxr*$^{fl/fl}$ mice.

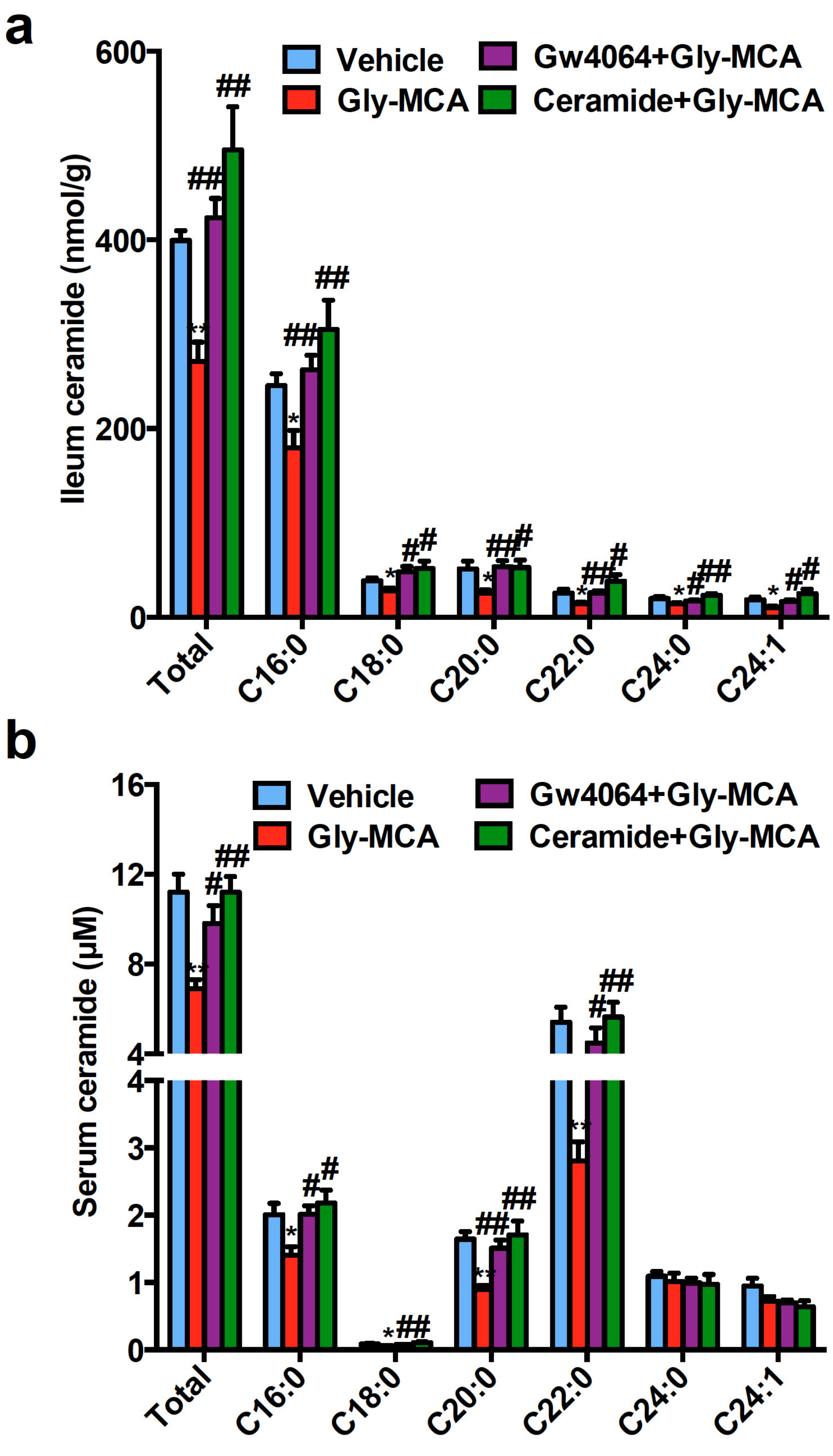

Supplementary Figure 11. Gly-MCA decreases ileum and serum ceramide levels.
(a,b) Ileum (a) and serum (b) ceramide levels in vehicle-, Gly-MCA-, Gw4064+Gly-MCA-, and ceramide+Gly-MCA-treated mice concurrently fed a HFD for 5 weeks. *n* = 5 mice per group. Data are presented as mean ± sd. One-way ANOVA with Tukey's correction. **P* < 0.05, ***P* < 0.01 compared to vehicle-treated mice. #*P* < 0.05, ##*P* < 0.01compared to Gly-MCA-treated mice.

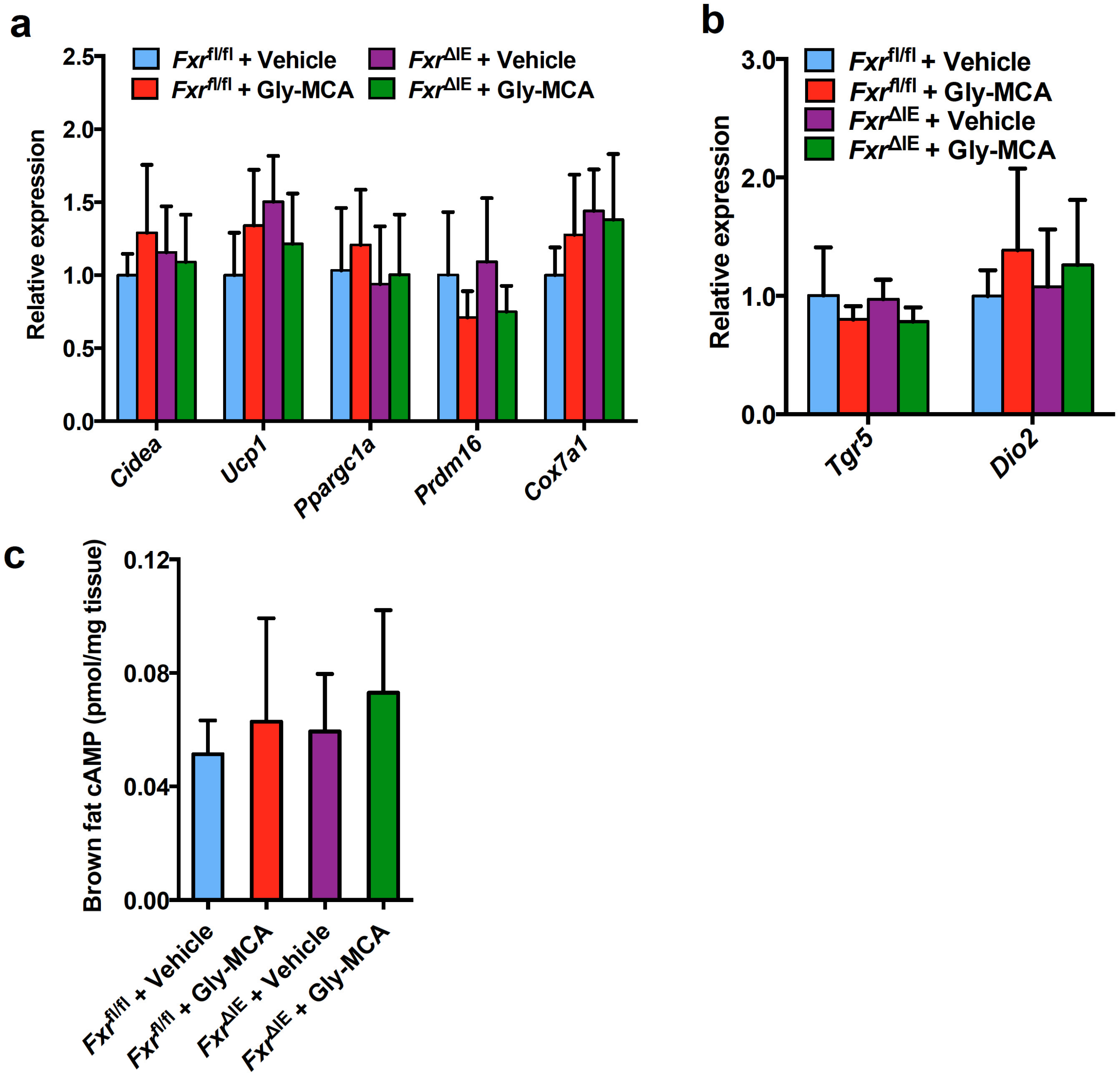

Supplementary Figure 12. Gly-MCA does not affect brown fat biogenesis.

Fxr$^{fl/fl}$ and *Fxr*$^{\Delta IE}$ mice fed a HFD for 8 weeks were concurrently treated with vehicle or Gly-MCA .

(a) Expression of brown fat thermogenic genes. n = 5 mice per group. Data are presented as mean ± sd. One-way ANOVA with Tukey's correction.

(b) The expression of mRNAs encoded by *Tgr5* and *Dio2* in brown fat. Expression was normalized to 18S RNA. n = 5 mice per group. Data are presented as mean ± sd. One-way ANOVA with Tukey's correction.

(c) The cAMP levels in brown fat. n = 5 mice per group. Data are presented as mean ± sd. One-way ANOVA with Tukey's correction.

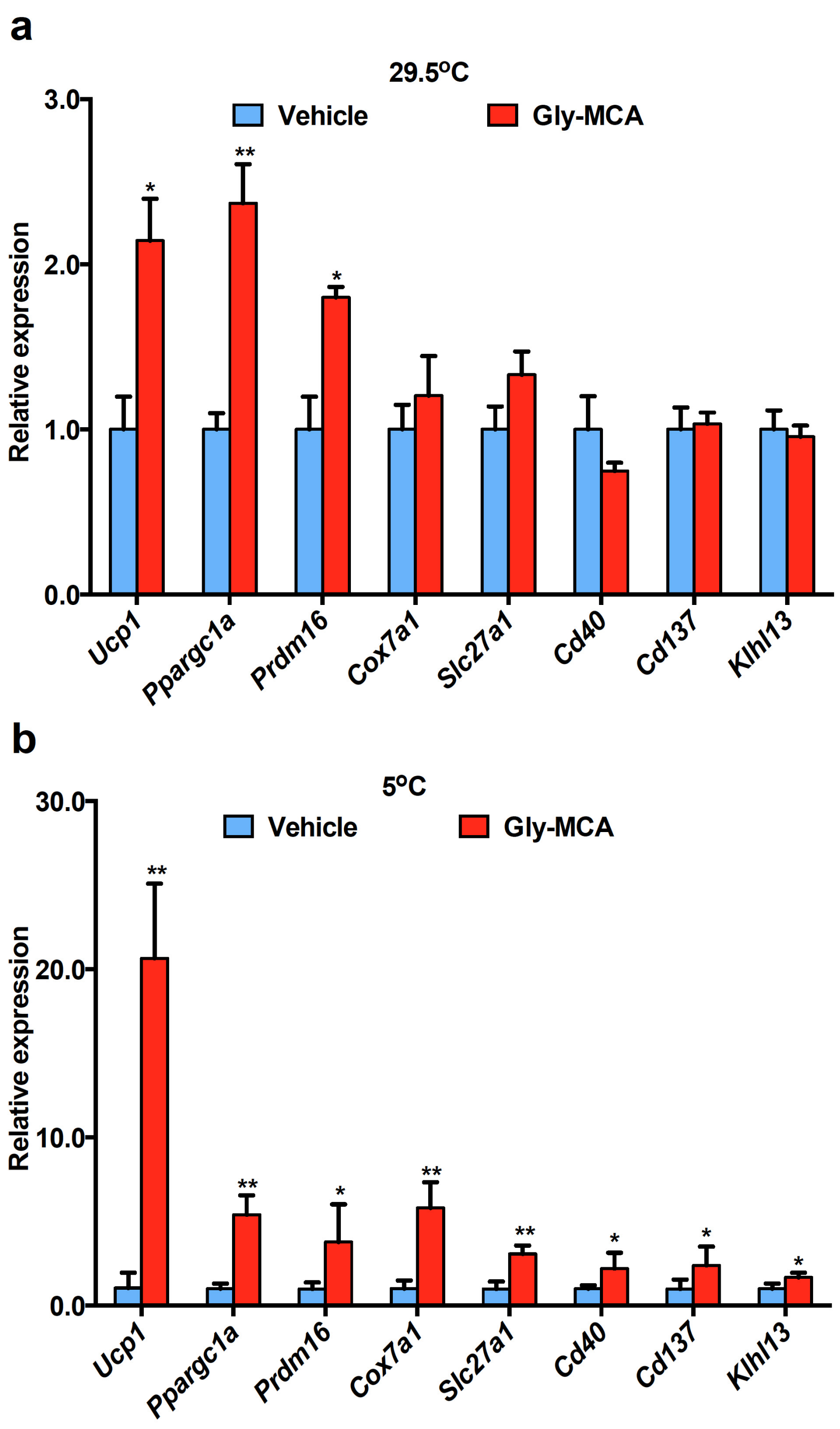

Supplementary Figure 13. Gly-MCA markedly promotes beige fat thermogenesis at both thermoneutral and cold temperature.

(a) mRNA levels of beige fat thermogenic genes in subcutaneous adipose tissue of vehicle- and Gly-MCA-treated mice fed a HFD at 29.5ºC for 2 weeks. $n = 5$ mice per group. Expression was normalized to 18S RNA. Data are presented as mean ± sd. Two-tailed Student's t-test. $*P < 0.05$, $**P < 0.01$ compared to vehicle-treated mice.

(b) mRNA levels of beige fat thermogenic genes in subcutaneous adipose tissue of vehicle- and Gly-MCA-treated mice fed a HFD at 22ºC for 5 days prior to at 5ºC for 1 day. Expression was normalized to 18S RNA. $n = 5$ mice per group. Data are presented as mean ± sd. Two-tailed Student's t-test. $*P < 0.05$, $**P < 0.01$ compared to vehicle-treated mice.

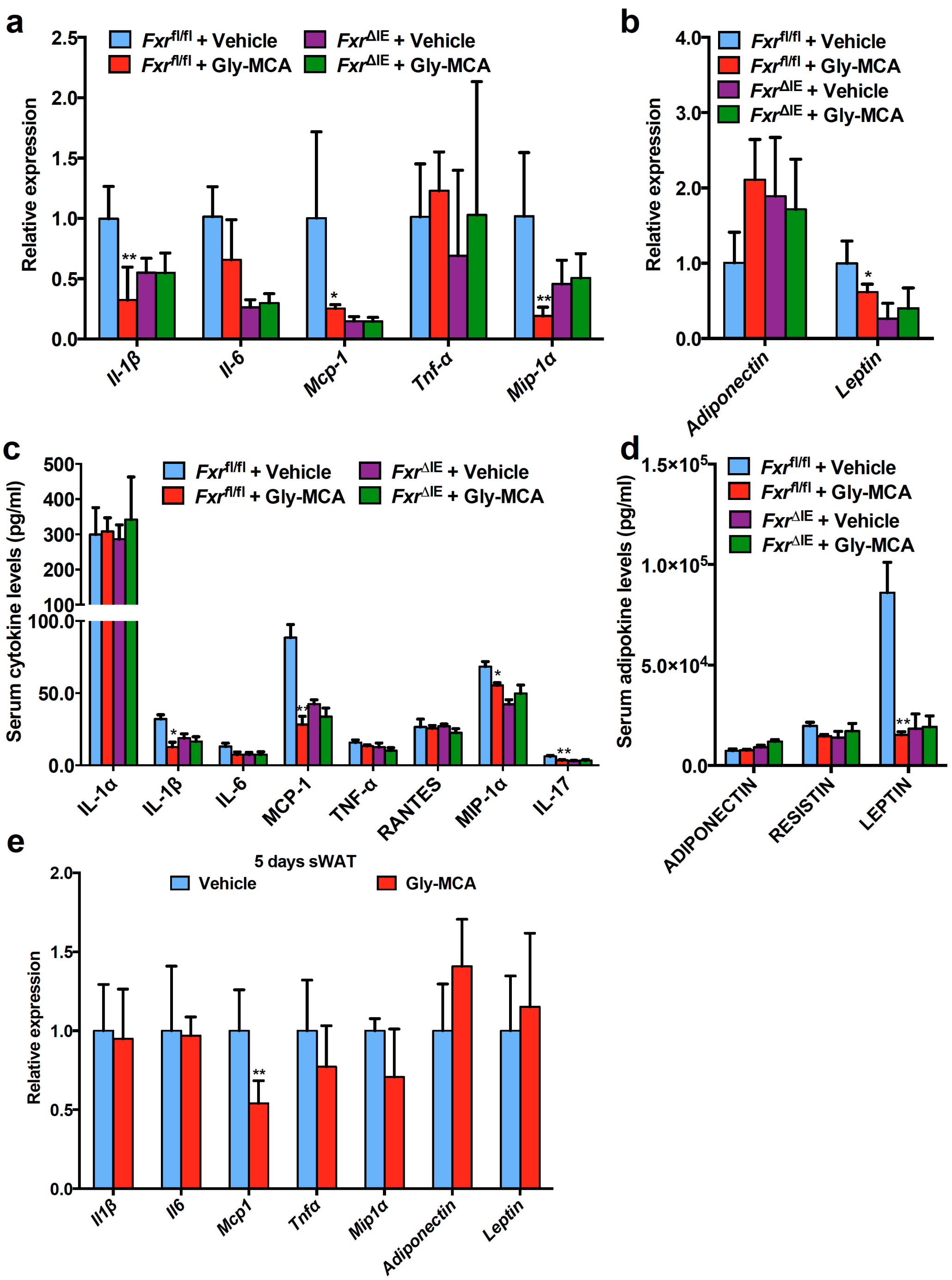

Supplementary Figure 14. Gly-MCA decreases adipose tissue and serum inflammatory cytokines mRNA levels and concentrations via the inhibition of intestinal FXR.

(a, b) mRNA levels of inflammatory cytokines related genes (a), adiponectin and leptin (b) in subcutaneous white adipose tissue. $Fxr^{fl/fl}$ and $Fxr^{\Delta IE}$ mice fed a HFD for 8 weeks were treated concurrently with vehicle or Gly-MCA. n = 5 mice per group. Data are presented as mean ± sd. One-way ANOVA with Tukey's correction. *$P < 0.05$, **$P < 0.01$ compared to vehicle-treated $Fxr^{fl/fl}$ mice.

(c, d) Serum cytokines levels (c) and serum adipokines levels (d). The $Fxr^{fl/fl}$ and $Fxr^{\Delta IE}$ mice fed a HFD for 8 weeks were concurrently treated with vehicle or Gly-MCA. n = 5 mice per group. Data are presented as mean ± sd. One-way ANOVA with Tukey's correction. *$P < 0.05$, **$P < 0.01$ compared to vehicle-treated $Fxr^{fl/fl}$ mice.

(e) mRNA levels of inflammatory cytokines related genes, adiponectin and leptin in subcutaneous adipose tissue of the mice with or without Gly-MCA treatment on a HFD for 5 days. $n = 5$ mice per group. Data are presented as mean ± sd. Two-tailed Student's t-test. $^{**}P < 0.01$ compared to vehicle-treated mice.

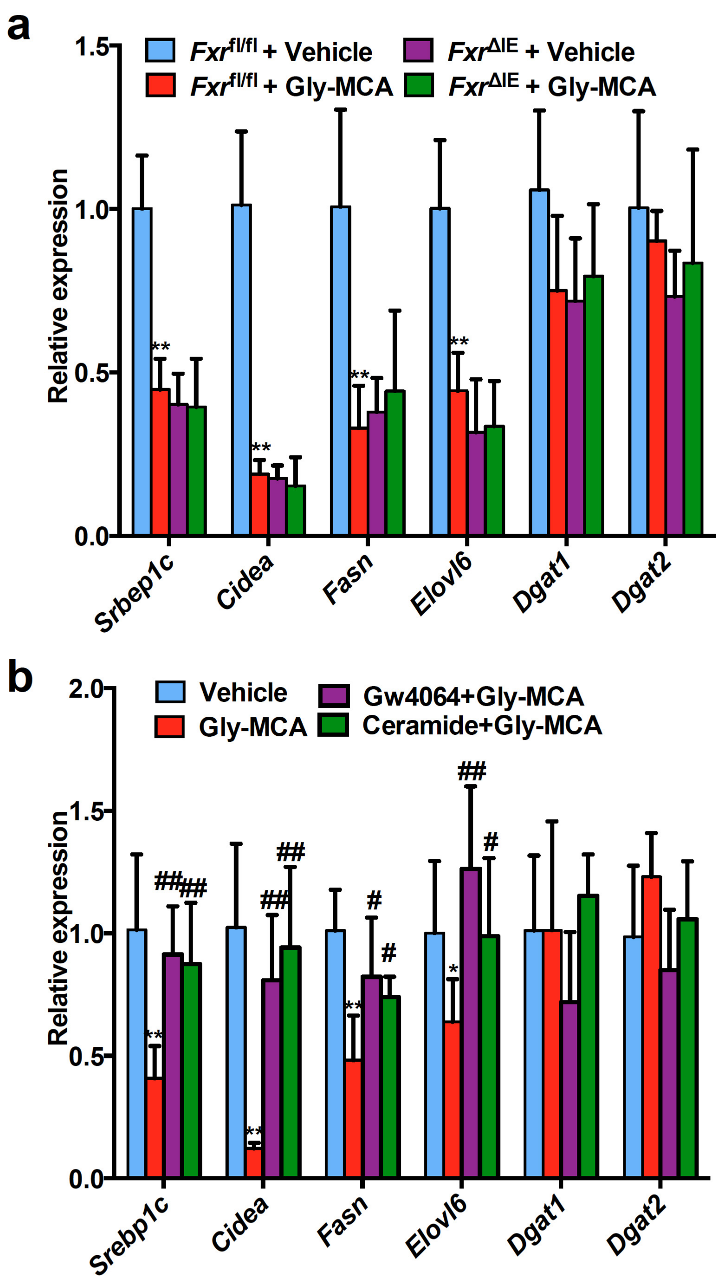

Supplementary Figure 15. Gly-MCA regulates the hepatic SREBP1C-CIDEA pathway through the intestinal FXR-ceramide axis.

(a) Hepatic mRNA levels of fatty acid synthesis and triglyceride synthesis related genes of vehicle- and Gly-MCA-treated $Fxr^{fl/fl}$ and $Fxr^{\Delta IE}$ mice concurrently fed a HFD for 8 weeks. n = 5 mice per group. Data are presented as mean ± sd. One-way ANOVA with Tukey's correction. $^{**}P < 0.01$ compared to vehicle-treated $Fxr^{fl/fl}$ mice.

(b) Hepatic mRNA levels of fatty acid synthesis and triglyceride synthesis related genes of vehicle-, Gly-MCA-, Gw4064+Gly-MCA-, and ceramide+Gly-MCA-treated mice concurrently fed a HFD for 5 weeks. n = 5 mice per group. Data are presented as mean ± sd. One-way ANOVA with Tukey's correction. $^{*}P < 0.05$, $^{**}P < 0.01$ compared to vehicle-treated mice. $\#P < 0.05$, $\#\#P < 0.01$ compared to Gly-MCA-treated mice.

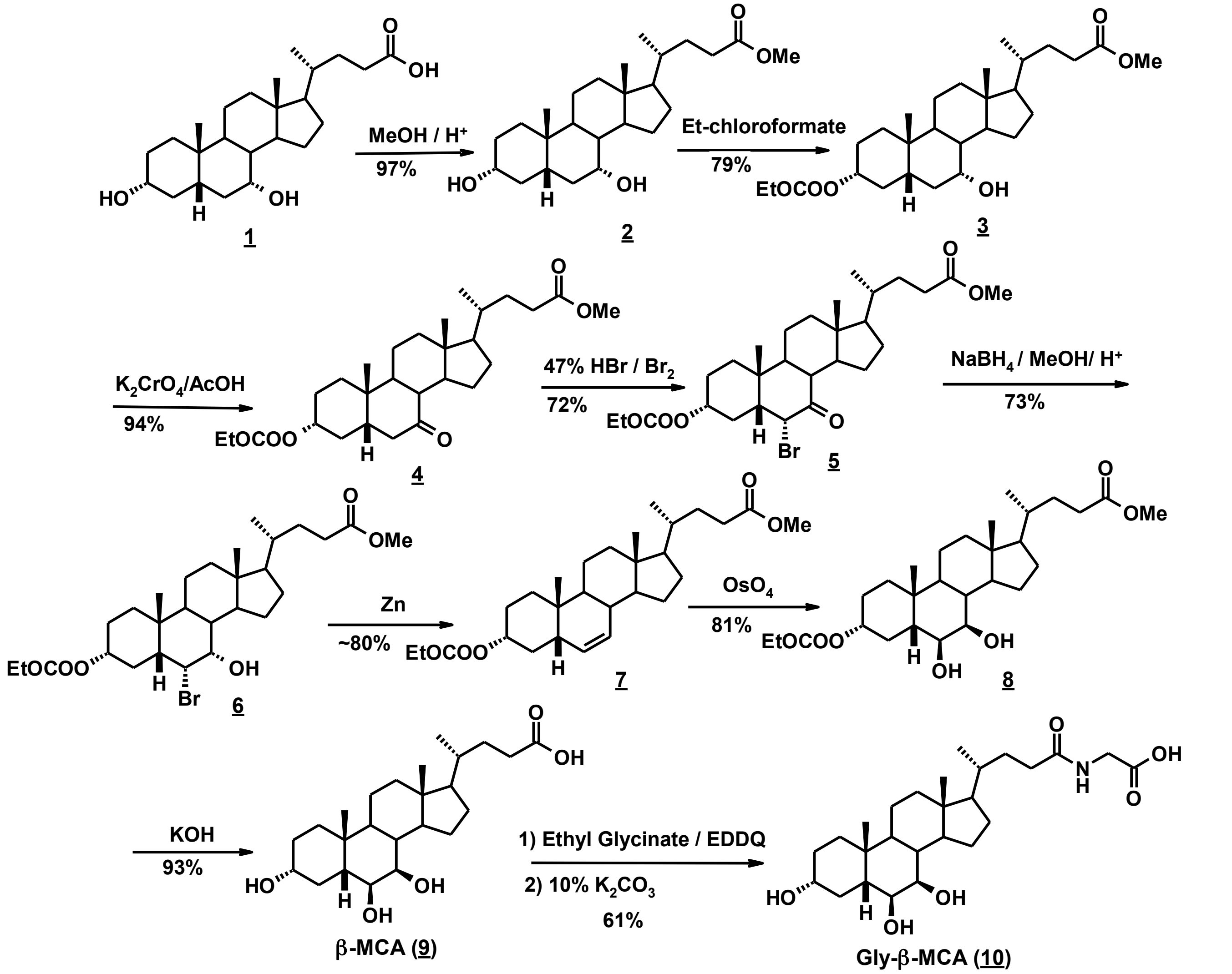

Supplementary Figure 16. The scheme for the synthesis of Gly-MCA.

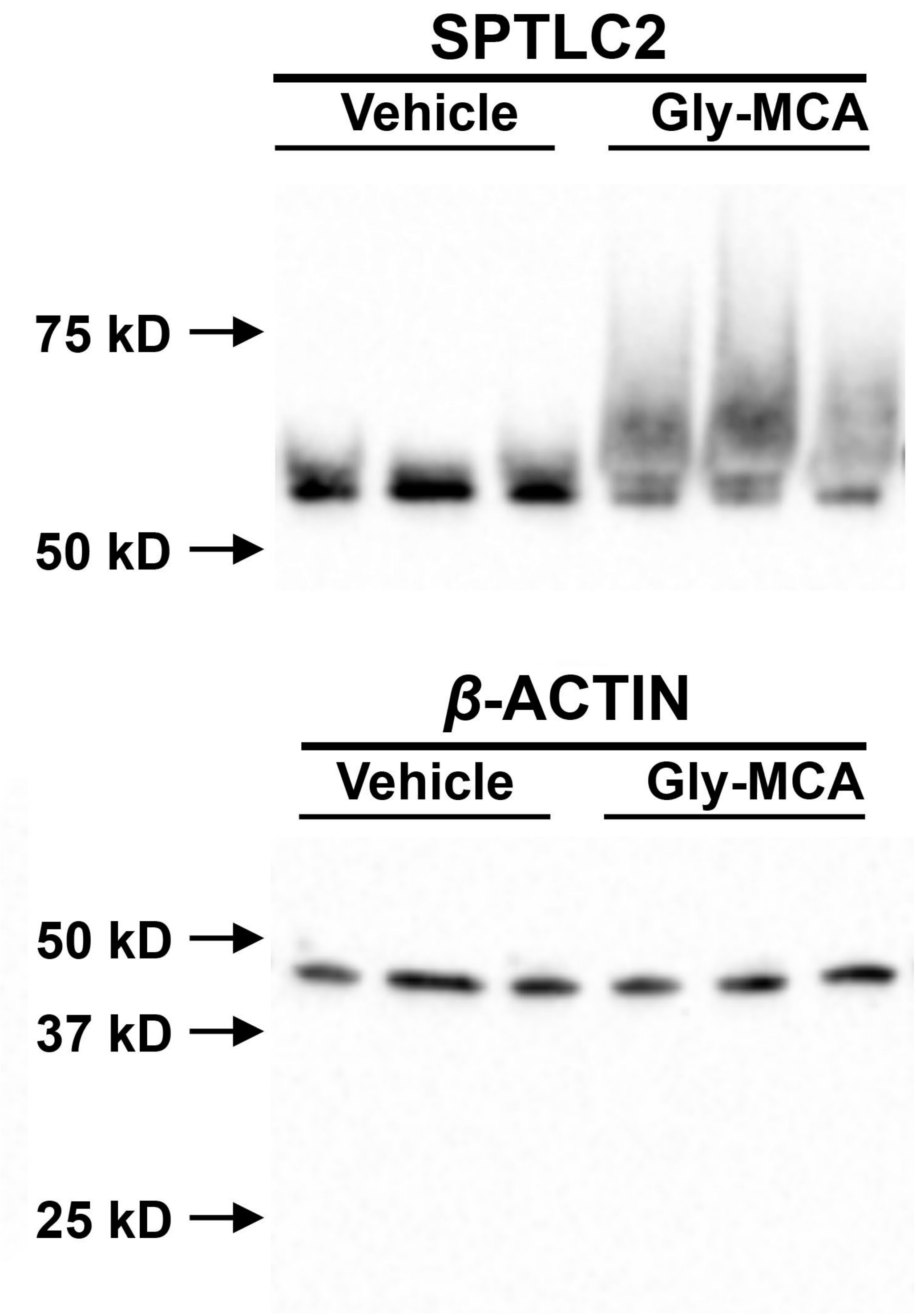

Supplementary Figure 17. Full western blot gel panel from Figure 3k.

Supplementary Table 1. ICM docking score data into human FXR-LBD in the agonist conformation (PDB 1OT7; Mi et al., 2003)

Compound	Score
3dCDCA	- 39.03
CDCA	- 42.8
α-TMCA	- 23.13
T-β-MCA	- 27.85
Gly-MCA	- 31.7
β-MCA	- 22.1

Supplementary Table 2. Demographic characteristics of the subjects

Characteristics	Lean	Obese
Sex, n (*%*)		
Male	21 (58.3)	18 (60)
Female	15 (41.7)	12 (40)
Age, *y*		
Mean (Sem)	47.9 (12.1)	49.7 (11.3)
Range	18.0-67.0	19.0-67.0
Body mass index, *kg/m*^{2}		
Mean (Sem)	21.9 (1.9)	27.3 (1.7)**
Range	17.9-24.4	25.4-32.1

** $P < 0.01$ compared to Lean.

Supplementary Table 3. Primers used for qPCR

Mouse primers	Sequence
18S FWD	5'- ATTGGAGCTGGAATTACCGC -3'
18S REV	5'- CGGCTACCACATCCAAGGAA -3'
Fxr FWD	5'- TGGGCTCCGAATCCTCTTAGA -3'
Fxr REV	5'- TGGTCCTCAAATAAGATCCTTGG -3'
Shp FWD	5'- TCTGCAGGTCGTCCGACTATTC -3'
Shp REV	5'- AGGCAGTGGCTGTGAGATGC -3'
Cyp7a1 FWD	5'- AACAACCTGCCAGTACTAGATAGC -3'
Cyp7a1 REV	5'- GTGTAGAGTGAAGTCCTCCTTAGC -3'
Cyp8b1 FWD	5'- CTAGGGCCTAAAGGTTCGAGT -3'
Cyp8b1 REV	5'- GTAGCCGAATAAGCTCAGGAAG -3'
Fgf15 FWD	5'- GCCATCAAGGACGTCAGCA -3'
Fgf15 REV	5'- CTTCCTCCGAGTAGCGAATCAG -3'
Srebp1c FWD	5'- GGAGCCATGGATTGCACATT-3'
Srebp1c REV	5'- GCTTCCAGAGAGGAGGCCAG -3'
Cidea FWD	5'- TGACATTCATGGGATTGCAGAC -3'
Cidea REV	5'- GGCCAGTTGTGATGACTAAGAC -3'
Fasn FWD	5'- AAGTTGCCCGAGTCAGAGAACC -3'
Fasn REV	5'- ATCCATAGAGCCCAGCCTTCCATC -3'
Elovl6 FWD	5'- GAAAAGCAGTTCAACGAGAACG -3'
Elovl6 REV	5'- AGATGCCGACCACCAAAGATA -3'
Dgat1 FWD	5'- GACGGCTACTGGGATCTGA -3'
Dgat1 REV	5'- TCACCACACACCAATTCAGG -3'
Dgat2 FWD	5'- CGCAGCGAAAACAAGAATAA-3'
Dgat2 REV	5'- GAAGATGTCTTGGAGGGCTG-3'

Sptlc1 FWD	5'- CGAGGGTTCTATGGCACATT-3'
Sptlc1 REV	5'- GGTGGAGAAGCCATACGAGT -3'
Sptlc2 FWD	5'- TCACCTCCATGAAGTGCATC -3'
Sptlc2 REV	5'- CAGGCGTCTCCTGAAATACC -3’
Sptlc3 FWD	5'- ACACAATCCTAAGACCCAGCA -3’
Sptlc3 REV	5'- AGACTGGCTTATCCTCAGCATA -3’
Degs1 FWD	5'- AATGGGTCTACACGGACCAG -3’
Degs1 REV	5'- TGGTCAGGTTTCATCAAGGAC -3’
Degs2 FWD	5'- AAGCCAATGGACCACAAACT -3’
Degs2 REV	5'- TGCTTGGAGAGCCCTTCTAAT -3’
Cers2 FWD	5'- AAGTGGGAAACGGAGTAGCG-3’
Cers2 REV	5'- ACAGGCAGCCATAGTCGTTC -3’
Cers4 FWD	5'- GGATTAGCTGATCTCCGCAC -3’
Cers4 REV	5'- CCAGTATGTCTCCTGCCACA -3’
Cers5 FWD	5'- CTTCTCCGTGAGGATGCTGT-3’
Cers5 REV	5'- GTGTCATTGGGTTCCACCTT -3’
Cers6 FWD	5'- AAGCCAATGGACCACAAACT -3’
Cers6 REV	5'- TGCTTGGAGAGCCCTTCTAAT -3’
Smpd1 FWD	5'- GTTACCAGCTGATGCCCTTC -3’
Smpd1 REV	5'- AGCAGGATCTGTGGAGTTG -3’
Smpd2 FWD	5'- AGCAGGATCTGTGGAGTTG -3’
Smpd2 REV	5'- CTCCAGCCATGAAGCTCAAC -3’
Smpd3 FWD	5'- CCTGACCAGTGCCATTCTTT -3’
Smpd3 REV	5'- AGAAACCCGGTCCTCGTACT -3’
Smpd4 FWD	5'- ACCTGGCCCTCAATCCATTTG -3’
Smpd4 REV	5'- ATAGGCACAGTCCGAAGTACG -3’

Ucp1 FWD	5'- GTGAACCCGACAACTTCCGAA -3’
Ucp1 REV	5'- TGCCAGGCAAGCTGAAACTC -3’
Ppargc1a FWD	5'- TATGGAGTGACATAGAGTGTGCT -3’
Ppargc1a REV	5'- CCACTTCAATCCACCCAGAAAG -3’
Prdm16 FWD	5'- CCACCAGCGAGGACTTCAC -3’
Prdm16 REV	5'- GGAGGACTCTCGTAGCTCGAA -3’
Cox7a1 FWD	5'- CAGCGTCATGGTCAGTCTGT -3’
Cox7a1 REV	5'- AGAAAACCGTGTGGCAGAGA -3’
Slc27a1 FWD	5'- CTGGGACTTCCGTGGACCT -3’
Slc27a1 REV	5'- TCTTGCAGACGATACGCAGAA -3’
Cd40 FWD	5'- TTGTTGACAGCGGTCCATCTA -3’
Cd40 REV	5'- CCATCGTGGAGGTACTGTTTG -3’
Cd137 FWD	5'- CGTGCAGAACTCCTGTGATAAC -3’
Cd137 REV	5'- GTCCACCTATGCTGGAGAAGG -3’
Klhl13 FWD	5'- CATTTCACTTCCAAGGCACA -3’
Klhl13 REV	5'- CTGTCTGCTCAACCCTGAGA -3’
Tgr5 FWD	5'- CTGTGTGAGATCCGCCGAC -3’
Tgr5 REV	5'- CGACGCTCATAGGCCAAGA -3’
Dio2 FWD	5'- CAGCTTCCTCCTAGATGCCTA -3’
Dio2 REV	5'- CTGATTCAGGATTGGAGACGTG -3’
Glp1 FWD	5'- GCACACAGTGCCCTAACCT -3’
Glp1 REV	5'- TTTAGCTGCTTCCGTGGTCTT -3’
Pxr FWD	5'- TAGGGACCTGCCTATTGAGGA -3’
Pxr REV	5'- CCGTTTCCGTGTCGAACATC -3’
Il-1β FWD	5'- GCAACTGTTCCTGAACTCAACT -3’
Il-1β REV	5'- ATCTTTTGGGGTCCGTCAACT -3’

Il-6 FWD	5'- TAGTCCTTCCTACCCCAATTTCC -3’
Il-6 REV	5'- TTGGTCCTTAGCCACTCCTTC -3’
Mcp-1 FWD	5'- TTAAAAACCTGGATCGGAACCAA -3’
Mcp-1 REV	5'- GCATTAGCTTCAGATTTACGGGT -3’
Tnf-α FWD	5'- CCACCACGCTCTTCTGTCTAC -3’
Tnf-α REV	5'- AGGGTCTGGGCCATAGAACT -3’
Mip-1α FWD	5'- TTCTCTGTACCATGACACTCTGC -3’
Mip-1α REV	5'- CGTGGAATCTTCCGGCTGTAG -3’
Adiponectin FWD	5'- TGTTCCTCTTAATCCTGCCCA -3’
Adiponectin REV	5'- CCAACCTGCACAAGTTCCCTT -3’
Leptin FWD	5'- GAGACCCCTGTGTCGGTTC -3’
Leptin REV	5'- CTGCGTGTGTGAAATGTCATTG -3’
**Rat primers**	**Sequence**
β-actin FWD	5'- GAGACCTTCAACACCCCAGCC -3’
β-actin REV	5'- TCGGGGCATCGGAACCGCTCA -3’
Ucp1 FWD	5'- CCAAAGTCCGCCTTCAGA -3’
Ucp1 REV	5'- TCATCAAGCCAGCCGAGA -3’
Ppargc1a FWD	5'- TGTCACCACCGAAATCCTTAT -3’
Ppargc1a REV	5'- TCAGACTCCCGCTTCTCATAC -3’
Prdm16 FWD	5'- AGAAGCACGAACACGAAGGT -3’
Prdm16 REV	5'- CCTCAGGCCGTTTGTCCATT -3’
Cox7a1 FWD	5'- GAGTGGCACAGAAGCAGA -3’
Cox7a1 REV	5'- TTGGGTTCTCAAGAGGTTAG -3’
Slc27a1 FWD	5'- TTCTGCGAGAACCCGTGAG -3’
Slc27a1 REV	5'- TCAGAACAGAGAGGCCAAAGAG -3’

Cd40 FWD	5'- CCGGTCGGATTCTTCTCCAAT -3’
Cd40 REV	5'- CCTCAGGGGGTAAGACCTCA -3’
Cd137 FWD	5'- TAAGAATGGGACCAAGGAGAAAGAC -3’
Cd137 REV	5'- GCACAGAGAACCAGAGAATGATGAA -3’
Klhl13 FWD	5'- TGTTGCTGCTACTCTACTCTGTC -3’
Klhl13 REV	5'- CCTCTTCCACAAGTGATCCTG -3’
**Human primers**	**Sequence**
18S FWD	5'- GATATGCTCATGTGGTGTTG -3'
18S REV	5'- AATCTTCTTCAGTCGCTCCA -3'
Fxr FWD	5'- AACCATACTCGCAATACAGCAA-3'
Fxr REV	-3'ACAGCTCATCCCCTTTGATCC-3'
Shp FWD	5'- CCCCAAGGAATATGCCTGCC-3'
Shp REV	5'-TAGGGCGAAAGAAGAGGTCCC-3'
Fgf19 FWD	5'-CCAGAAGACAGGCAGTAGT-3'
Fgf19 REV	5'-CTGGAGGGATTTGGGAAGG-3'

Supplemental reference

Mi, L.Z., Devarakonda, S., Harp, J.M., Han, Q., Pellicciari, R., Willson, T.M., Khorasanizadeh, S., and Rastinejad, F. (2003). Structural basis for bile acid binding and activation of the nuclear receptor FXR. Mol Cell *11*, 1093-1100.
